# Clinical Evidence on Particle Radiation, DNA Damage Response Inhibitors, and Immunotherapy for Mismatch Repair-Proficient Rectal Cancer

**DOI:** 10.3390/cancers18040652

**Published:** 2026-02-17

**Authors:** Cristian J. Salazar-Vilches, Daniel K. Ebner, Jake A. Kloeber, Sonja Dragojevic, Jasvinder Singh, Michael Haddock, Yasamin Sharifzadeh, Alexander D. Sherry, Krishan R. Jethwa, Christopher L. Hallemeier, Kenneth Merrell, Robert W. Mutter, Zhenkun Lou, Cameron M. Callaghan

**Affiliations:** 1 Department of Radiation Oncology, Mayo Clinic, Rochester, MN 55905, USA; salazarvilches.cristian@mayo.edu (C.J.S.-V.);; 2 Department of Oncology, Mayo Clinic, Rochester, MN 55905, USA

**Keywords:** rectal cancer, particle therapy, immunotherapy, DNA damage response inhibitors

## Abstract

Background/Objectives: We performed a systematic review of the current clinical and preclinical literature on the use of particle therapy, DDRi, and/or immunotherapy, specifically for pMMR colorectal cancer. Methods: A systematic review of the literature published between 2014 and 2025 was conducted across major databases. Studies were included if they examined particle radiotherapy (e.g., proton, alpha, carbon) either alone or in combination with DDRi and/or immune checkpoint inhibitors (ICI), or X-ray radiotherapy (XRT) in combination with DDRi/ICI. Results: In total, 133 studies met the inclusion criteria, including 62 clinical studies. Clinically, particle therapies show excellent local control and normal tissue sparing, with manageable toxicity profiles. Trials with any form of radiation and DDRi are few, potentially owing to toxicity concerns. ICI combinations showed promising efficacy with XRT, with no randomized trials comparing them to particle radiation. Conclusions: Particle radiation and/or DDRi have significant preclinical evidence of immunostimulatory effects in pMMR rectal adenocarcinoma and increase response rates to immunotherapy (presented in the companion manuscript). Despite strong preclinical evidence and rationale, clinical trials including all three modalities are scarce. Existing evidence suggests a potential benefit based on extrapolation from photon-based studies and supports prospective evaluation with careful attention to treatment-related toxicity, which remains a concern.

## Simple Summary

Patients with mismatch repair-deficient (dMMR) rectal adenocarcinomas have demonstrated significant complete response rates to PD-1 inhibitor monotherapy, potentially allowing them to avoid chemotherapy, radiation, and surgery. However, >95% of patients are mismatch repair proficient (pMMR) and have so far not responded to immunotherapy in clinical trials. There is growing evidence that both high linear energy transfer (LET) particle radiation and DNA damage response inhibitors (DDRi) may increase the response of pMMR rectal cancer to immunotherapy and are ready for clinical translation.

## 1. Introduction

Colorectal cancer is a leading cause of cancer-related mortality, with an increasing incidence among younger patients [1,2]. While mismatch repair deficiency (dMMR) confers a substantial mutational burden and robust response to immunotherapy, over 95% of rectal cancer patients are MMR proficient (pMMR) [3] and do not respond as well to immunotherapy alone [4].

Standard treatments for locally advanced pMMR rectal adenocarcinoma include radiotherapy (RT), chemotherapy, and surgery or wait-and-watch surveillance for patients with a complete clinical response after total neoadjuvant therapy (TNT) [5]. TNT improves organ preservation rates and disease outcomes. However, with standard TNT, only ~20–40% of patients achieve a complete clinical response, and those opting for non-operative management (NOM) remain at risk of local and distant recurrence. Enhancing the response of pMMR disease to immunotherapy could improve outcomes, reduce toxicity, and increase the proportion of patients eligible for NOM [6,7].

Defects in the DNA damage response (DDR), and specifically MMR and homologous recombination (HR), increase tumor mutation burden, genomic instability, neoantigens, and potentially sensitize tumors to ICI [8,9,10]. This has been demonstrated most dramatically by the complete response rates of dMMR rectal cancers to PD-1 inhibition [11]. Mutations leading to DDR defects may also predict synthetic lethality with DDRi, as seen in BRCA mutants and PARP inhibitors. Inactivation of DDR pathways (via loss-of-function mutations or pharmacological inhibition) can further enhance immune-based therapies by amplifying DNA damage signaling and antitumor immune responses [8,12,13].

Linear energy transfer (LET) describes the average energy deposited per unit path length along a charged particle track (LET = dE/dℓ; typical units keV/µm). High-LET commonly refers to ≳10 keV/µm, in contrast to low-LET photons and most clinical proton beams, although the proton LET increases as the depth increases (Bragg peak). High–linear energy transfer radiation produces more complex, clustered double-strand breaks (DSBs) than low-LET radiation such as conventional X-ray radiotherapy (XRT). Repair of these complex DSBs often relies on error-prone repair pathways, promoting genomic instability and generating potent immunogenic stress signals [14,15,16]. Additionally, high-LET radiation combined with DDRi has shown improved radiosensitization compared to x-rays [16,17,18] and elicits a more robust immune response [19].

In this review, we systematically examined the clinical evidence on the interplay between RT, immunotherapy, and DDRi in rectal cancer (with preclinical evidence presented in a companion manuscript). We explored the rationale and advantages of high- versus low-LET radiation and evaluated the efficacy and potential of combination therapies in enhancing therapeutic outcomes, with a focus on pMMR rectal cancer.

## 2. Materials and Methods

### 2.1. Literature Search

**Figure 1:**
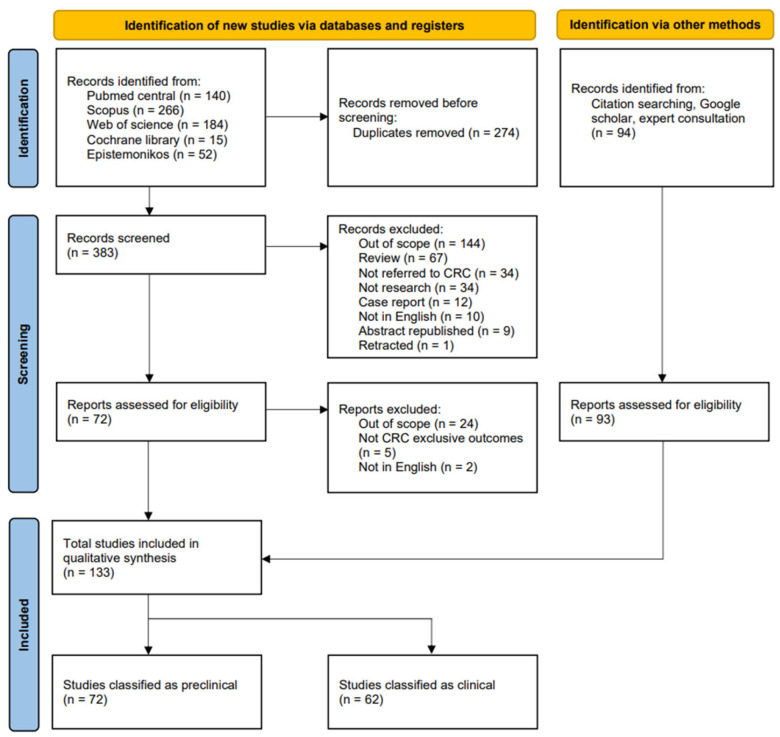
PROSPERO flowchart for Systematic Literature Search for both clinical (current manuscript) and preclinical data (companion manuscript). The collection, inclusion, and exclusion process of the systematic literature search adhered to PROSPERO guidelines https://www.crd.york.ac.uk/PROSPEROFILES/627912_STRATEGY_20250208.pdf, accessed on 13 February 2026 (registration #CRD42024627912 at the PROSPERO website).

Studies were included if they examined particle radiotherapy (e.g., proton, alpha, carbon), either alone or in combination with DDRi and/or immune checkpoint inhibitors (ICI). Studies on XRT were included in the literature search and systematic review if combined with DDRi or ICI. As many relevant studies are single-arm non-randomized trials, trials using XRT without DDRi or ICI are cited as historical comparators (but were not included in the literature search and systematic review). We searched PubMed Central, Cochrane Library, Scopus, Epistemonikos, Web of Science, and Google Scholar for articles published between 1 January 2014 and 31 August 2025 (date of last search) using combinations of terms related to rectal cancer and high-LET radiation therapies. Search terms included variations of “rectal cancer,” “colorectal cancer,” “rectal carcinoma,” “colorectal carcinoma,” “rectal tumor,” and “colorectal tumor,” combined with terms such as “high LET radiation,” “high linear energy transfer radiation,” “proton therapy,” “carbon ion radiation therapy,” “CIRT,” and “diffusing alpha particle radiation therapy” or “DaRT.” For studies examining combination therapies, additional terms included “immunotherapy,” “PD-1,” “PD-L1,” “CTLA4,” “immune checkpoint inhibitors,” “DNA damage response inhibitors,” “PARP inhibitors,” “ATR inhibitors,” “ATM inhibitors,” “DNA-PK inhibitors,” “WEE1 inhibitors,” “CHK1 inhibitors,” and “CHK2 inhibitors” (including “inhibition” instead of “inhibitors”). As clinical particle-RT combination studies remain limited, we also included key photon-based chemoradiotherapy+ ICI/DDRi trials to contextualize clinical signals of RT-enabled immune priming as indirect clinical evidence. The search strategies were adapted for each database. The PICO search strategy, specific search strategies, and keywords for pMMR status identification are presented in Supplementary File. References cited in the included studies were manually reviewed to identify additional relevant articles. Two independent investigators (C.C. and C.S.) screened titles, abstracts, and full-text articles for inclusion. Studies were eligible if they investigated high-LET radiation, either as monotherapy or in combination with immunotherapy or DDRi, in preclinical colorectal cancer. The search was limited to articles published in English. Discrepancies were resolved through consensus. This systematic review adhered to the Preferred Reporting Items for Systematic Reviews and Meta-Analyses (PRISMA; Figure 1). The review search protocol can be accessed at (https://www.crd.york.ac.uk/PROSPEROFILES/627912_STRATEGY_20250208.pdf, accessed on 13 February 2026) or review registration # CRD42024627912 on the PROSPERO website.

### 2.2. Review and 4

Clinical studies were categorized based on the treatment modality: conventional X-ray radiotherapy (XRT) or particle radiation (proton beam therapy “PBT” or carbon-ion radiation therapy “CIRT”), alone or in combination with ICI or DDRi. Clinical trial data included study design, inclusion criteria, dose and fractionation, clinical disease setting (locally advanced, recurrent, or metastatic CRC/rectal cancer), and outcomes, such as local control (LC)/Local Failure (LF), progression-free survival (PFS), overall survival (OS), distant metastasis-free survival (DMFS)/distant failure (DF), response rates, and acute/late toxicity (> or <3 months).

Comparisons with conventional X-ray irradiation have been summarized in this report. Clinical studies were grouped according to treatment strategies and disease settings. A meta-analysis was not conducted because of the heterogeneity of the study designs and endpoints of clinical studies.

## 3. Results

### 3.1. Clinical Evidence

PBT vs. XRT for Locally Advanced Rectal Cancer (LARC)

It is well established that PBT plans can spare more normal tissue than XRT plans based on dosimetry alone [20,21], and specialized PBT techniques may improve sparing even more [22]. However, clinical data on efficacy and toxicity outcomes are limited, especially in randomized clinical trials (Table 1).

The PRORECT trial (NCT04525989) is a randomized phase 2 trial of PBT vs. XRT followed by CAPEOX x4 cycles and either surgery or wait-and-watch (WW) surveillance. In their preliminary report, surgical complications were comparable between XRT and PBT (N = 10 per cohort), and it was too early to assess the primary endpoint of preoperative G2–5 GI toxicity [23].

A retrospective study by Lin et al. [24] focused primarily on comparing long-course chemoradiation (LCCRT) followed by delayed surgery vs. short-course radiation (SCRT) followed by chemotherapy and then surgery. However, among the SCRT patients, 14.8% received PBT, while the rest were treated with XRT (N = 122 all SC patients, N = 104 XRT, and N = 18 PBT), and some comparisons were made between RT modalities. All SC patients had better pathologic complete response (pCR) and near-pathologic complete response (npCR) rates, with longer delays between SC radiation and surgery. The rates of pCR/npCR were 12.1% and 19.0% for short interval < vs. >21.9%/23.4% for delayed (< vs. >16 weeks). However, this was even more pronounced in patients treated with PBT, with pCR/npCR rates of 0/25% for the short interval vs. 30/40% for the delayed interval. Interestingly, in patients with CEA > 7 ng/mL (who had worse disease outcomes overall), PBT had higher rates of npCR (40%) compared to only 7.8% for all SC patients. For patients with baseline CEA levels < 7 ng/mL, the npCR rates were similar to those of XRT and PBT (~30%). Overall, this study suggests that SC PBT may be especially effective in patients with higher baseline CEA levels, and PBT may benefit more from delaying the time from SC to surgery by >16 weeks. They also noted that PBT seemed to be more effective for T4 disease, although they did not present specific data to demonstrate this. Interestingly, these results come with the caveats of a retrospective study, non-uniform chemotherapy regimens, and a relatively low number of patients in the PBT cohort.

PBT for Locoregionally Recurrent Rectal Cancer (LRRC)

While heterogeneity of the recurrent patient population and lack of prospective randomized trials comparing PBT to XRT make definite conclusions difficult in the recurrent setting (Table 2), PBT has superior normal tissue sparing compared to XRT for re-irradiation of LRRC. PBT is well tolerated and has reasonable efficacy in this population compared to the historical results of XRT. There are a variety of dose/fractionation regimens utilized, BID vs. daily fractionation patterns, supine vs. prone treatment positions, and variable use of chemotherapy and surgery in previous reports. Patients who undergo surgery, older patients, and those with more PET avidity prior to PBT (and complete metabolic response afterwards) may have better outcomes [21,25,26,27,28,29]. Although randomized clinical trial data are lacking, given the consistent dosimetric OAR sparing of PBT and the limitations on dose-escalation based on prior RT and OAR constraints in this population, PBT is promising, with the optimal technique and regimen being an active area of study.

CIRT for Locoregionally Recurrent Rectal Cancer (LRRC)

For LRRC, CIRT has been studied prospectively in single-arm clinical trials in patients with and without prior RT histories, in technically and medically unresectable patients, and in several different dose/fractionation regimens [30,31,32,33,34,35,36,37,38,39,40,41,42,43,44,45] (Table 3). A few retrospective studies have compared XRT to CIRT [43,44] or PBT and CIRT [46,47], but with the unique dose/fractionation pattern widely adopted for CIRT, direct comparisons to PBT or XRT are difficult. CIRT has typically been delivered in 16 fractions at four fractions per week, whereas XRT re-irradiation studies have used BID treatments, SBRT dosing, or conventional dosing, more commonly employed concurrent chemotherapy, and more commonly involved intended immediate re-resection after re-irradiation. The toxicity and efficacy of CIRT monotherapy compare favorably to a combined modality therapy approach of re-irradiation (30 Gy/15 fractions) with concurrent chemotherapy followed by immediate re-resection + IOERT [43]. Two techniques have been explored that may help to make re-irradiation with CIRT safer and more feasible. One is the surgical placement of spacers between the uninvolved but close normal bowel and recurrent disease. Although this allows a full dose to be delivered without uncovering the target (at least with respect to the areas of tumor adjacent to the bowel as opposed to sacral nerves), it may have complications of unintentional tumor seeding and/or injury to the adherent bowel when inserting the spacer. To address this, Kimura et al. 2025 [42] studied an approach of no spacer, putting no dose constraints on the adjacent/adherent bowel, and having a planned resection, not of the recurrent tumor, but of the normal small bowel/rectum 3–8 weeks after CIRT. Regions of the bowel receiving >46 Gy were resected (LAR for the rectum and small bowel resection for the small bowel). Additionally, an omental pedicle flap was placed between the recurrent disease and bowel if the patient required additional radiotherapy in the future. Although the cohort was small (N = 12), the results were promising in terms of postoperative complications, GI toxicity, and efficacy.

#### 3.1.1. PBT/CIRT Radiotherapy for Oligometastatic Disease

Clinical studies of particle therapy for oligometastatic disease have included dose fractionation regimens ranging from conventional fractionation to SBRT using PBT [50,51,52,53] or CIRT [54,55,56] for oligometastases to the liver, lungs [56,57,58], or nodal recurrence [45,59,60,61] (Table 4). No randomized trials have compared the same dose/fractionation regimens between XRT, PBT, and/or CIRT, but a few retrospective studies have attempted to compare outcomes.

Lee et al. 2025 showed improved 2- and 5-year local control with CIRT compared to XRT (see Table 5) [60]. Although the authors used propensity score matching to attempt to balance patient/disease risk factors between the cohorts, patients receiving XRT had a larger median pre-treatment gross tumor volume and a greater percentage of patients with ≥3 LNs being treated than those receiving CIRT. The differences in dose/fractionation regimens make it difficult to interpret the differential efficacy between XRT and CIRT, although the authors provided evidence that CIRT was feasible and well tolerated. A multicenter retrospective study by Aibe et al. 2023 evaluated PBT and CIRT compared to XRT for the treatment of oligometastatic disease in the liver, lungs, or lymph nodes from any primary site of disease [61]. For the CRC cohort (48 out of 132 total patients), CIRT/PBT was associated with favorable local control in the liver, with a non-significant trend towards improved LC in the lungs and lymph nodes compared to extracted historical outcome data for XRT SBRT. There was also a trend towards improved LC with CIRT compared to PBT in oligometastatic liver disease, but this was not significant. Fukumitsu et al., 2025 examined CIRT and PBT for 1–3 liver oligometastases from any primary site that had a sizable CRC contingent (>50%) [62], but an analysis of LC between CIRT and PBT was not conducted.

Even with the same radiation modality, retrospective comparisons of different dose/fractionation regimens are challenging. Clinically, the dose/fractionation regimen for these patients is often chosen based on the balance between efficacy and safety. Proximity to sensitive organs at risk or a large-volume target often leads clinicians to choose a more fractionated regimen and/or to allow for under-coverage of the target in order to deliver treatment safely. The volume and number of lesions can affect both the patient’s baseline risk of local failure and the clinically feasible dose/fractionation schedule. Despite this, the available data on PBT and CIRT for oligometastatic disease demonstrate excellent tolerability (all studies reporting grade 3 toxicity of <5%) and local control, which is at least as good, if not superior to, historical XRT studies, with no prospective randomized controlled trial data for direct comparison. It should also be noted that because of the normal tissue sparing of CIRT/PBT, even if such a trial were to be conducted, there would be many patients who could only be safely treated with CIRT/PBT, as XRT would not be able to meet normal organ dose constraints.

#### 3.1.2. Combinations with DDRi and/or ICI

##### IR + DDRi

DNA-PKi peposertib has been studied with long-course CRT in LARC, but the study was concluded early due to excess toxicity [81]. The well-known radiotherapy toxicity experienced by patients with ataxia-telangiectasia syndrome results from germline mutations, resulting in the lack of a functional ATM protein and compromised DDR. Transient pharmacological inhibition of DDR proteins may offer a therapeutic benefit based on the patient’s DDR defects already present in their normal tissues and tumors, as well as the volumes being included in the radiotherapy field. This highlights the importance of the timing, dosage, type of radiation, and radiotherapy fields (i.e., whether large elective fields including a significant portion of normal tissue vs. gross disease only) being used for treatment when combining IR+DDRi. Alternatively, a dose escalation study of long-course CRT (LCCRT) with the PARP inhibitor veliparib, followed by surgery in LARC, showed acceptable tolerability and compatibility with capecitabine, with a 29% pCR rate [63].

##### IR + ICI

Several recent trials have combined IR with ICIs in the treatment of LARC, either concurrently with IR or during chemotherapy with TNT.

Studies focusing on LCCRT have examined both concurrent and sequential ICI during/after long-course chemoradiation. NRG-GI002 did not show a significant benefit of adding pembrolizumab (aPD-1) concurrently with and then continued after LCCRT, although this was after an initial six cycles of FOLFOX [74]. Comparatively, the NECTAR study showed that patients treated with LCCRT with capecitabine + tislelizumab (aPD-1) for three cycles (two concurrent and one after LCCRT) achieved a favorable pCR rate (40%) without any chemotherapy prior to RT + ICI [78]. The VOLTAGE study used LCCRT followed by nivolumab and then surgery 10–12 weeks after completion of LCCRT. This was favorable for historical controls (3-year RFS of 80% and pCR rate of 29%) and did so without a chemotherapy component. Additionally, a PD-L1 tumor proportion score ≥ 1 and elevated CD8+ T cell/effector regulatory T cell ratio correlated with treatment response, potentially providing an avenue for patient selection [75]. Similarly, the PANDORA single-arm trial examined LCCRT followed by durvalumab with surgery 10–12 weeks after completion of LCCRT. The pCR rate was 34.5% [76], potentially indicating a difference between aPD-1 and aPD-L1 expression in this setting.

The POLARSTAR study most directly addressed the question of whether ICI is best combined with LCCRT concurrently or sequentially. This trial randomized patients into three arms prior to surgery: LCCRT alone (control), LCCRT with concurrent tislelizumab (aPD-1) (group A), or LCCRT followed by tislelizumab (group B). This resulted in pCR rates of 14, 27, and 33% for the control, group A (concurrent), and group B (sequential), respectively [80], demonstrating a trend towards better response with sequential sequencing. This trial allowed the control arm to have surgery starting at 6 weeks post-CCRT, compared to a minimum of 8 weeks for groups A and B, somewhat complicating the interpretation of the results.

Alternatively, chemoimmunotherapy, as the first portion of TNT followed by LCCRT without concurrent immunotherapy, has also performed well. In a study by Xiao et al. 2024 [79], investigators examined CAPEOX x4 cycles with or without concurrent sintilimab (aPD-1) followed by LCCRT followed by surgery or NOM. CR (cCR+pCR) rates of 26.9 and 44.8% were achieved in the control and sintilimab arms, respectively. Li et al. 2024 examined initial CAPEOX+camrelizumab (aPD-1) followed by LCCRT and then CAPEOX alone, which achieved a pCR rate of 33.3% (with an additional 38% with major pathologic response) and a cCR rate of 48% [77].

Comparisons of SCRT and LCCRT were performed in the PRIME-RT trial. This phase 2 randomized trial compared SCRT (arm A) and LCCRT (arm B), followed by FOLFOX with durvalumab (aPD-L1) administered concurrently during both RT and FOLFOX. Patients then underwent surgery or NOM depending on whether they achieved cCR. At 6 months, the combined cCR+pCR rate was 67% with SCRT (arm A) and 48% after LCCRT (arm B), with pCR+sustained cCR rates of 61 and 38%, respectively, at 1-year post-treatment. Three of 39 patients had dMMR (two in arm B) [68]. Although early, this does give a potential signal that a short course may offer more synergy with aPD-L1 than LCCRT when given up front in TNT.

The TORCH trial was a phase 2 randomized trial that examined the effect of SCRT timing combined with chemoimmunotherapy in patients with pMMR/MSS LARC. Patients were randomized to either short-course RT (SCRT) followed by CAPEOX+ toripalimab (aPD-1) x6 cycles (group A) or the same regimen, but SCRT was delivered between cycles 2 and 3 of CAPEOX+toripalimab (group B) [66]. Subsequently, patients either underwent surgery or WW if they achieved a complete response. Both groups achieved a combined cCR or pCR (if the patient underwent surgery) of 54–56%, suggesting that the results were good compared to the historical TNT results; however, the timing of SCRT in these two regimens did not make a meaningful difference.

UNION is a phase 3 randomized trial of SCRT followed by CAPEOX+camrelizumab (aPD-1) or LCCRT followed by CAPEOX (two cycles each). All patients underwent surgery and received adjuvant CAPEOX+camrelizumab (aPD-1) or CAPEOX. The SCRT and CAPEOX+camrelizumab arms had a pCR rate of 39.8% compared to 15.3% in the LCCRT and CAPEOX alone arms [69]. As both LCCRT and SCRT, and the use or absence of camrelizumab, differed between the arms, the LCCRT arm essentially acted as a historical control with less chemotherapy than is commonly utilized currently. Nevertheless, this study provided more evidence of synergy with SCRT followed by chemoimmunotherapy as a promising TNT strategy. Further data supporting this strategy was provided by the SPRING-01 trial. A randomized phase 2 trial of LARC patients treated with SCRT followed by CAPEOX x6 with or without sintilimab (aPD-1) concurrent with CAPEOX 1 week later. Patients then underwent TME in all cases 2–3 weeks after TNT. The MMR status was roughly equal between the two arms with 84–88% pMMR, 2% dMMR, and 10–14% with an unknown status. The sintilimab arm had a significantly higher pCR rate than the control arm (29 vs. 16%, respectively) [82]. STELLAR II was a randomized phase 2 trial examining SCRT followed by CAPEOX x4 with or without sintilimab (aPD-1), followed by TME or NOM depending on cCR. Although FOLFOX x6 cycles were allowed, only 1 out of 214 patients received FOLFOX; therefore, no comparison was possible concerning whether CAPEOX or FOLFOX had better synergy with sintilimab. The rate of CR (cCR+pCR) was 45.5 vs. 25.0% in the sintilimab and control cohorts, respectively [70,83].

PD-L1 inhibitors have also shown activity in these settings, as demonstrated in the PRECAM trial. This was a single-arm phase 2 trial of MSS LARC treated with SCRT followed by CAPEOX+Envafolimab (aPD-L1) and TME 2 weeks after chemoimmunotherapy. While these patients may have had earlier-stage disease than other LARC trials, it demonstrated an impressive pCR rate of 62.5% [73]. The AVERECTAL trial showed a relatively good response to SCRT followed by FOLFOX with concurrent avelumab (aPD-L1), with 25% pCR and an additional 25% with near-pCR [72].

In a metastatic setting, Parikh et al. 2021 [65] conducted a phase 2 single-arm clinical trial of patients with metastatic CRC or PDAC (MSS) treated with cycles of Ipi/Nivo → Nivo with SBRT administered to sites of metastatic disease (24 Gy/3 fx) during the 2nd cycle of Ipi/Nivo. There was a trend towards increased resting NK cells in pretreatment biopsies in responders compared to non-responders. Although both XRT and PBT were used for SBRT, they were not directly compared. A significant number of patients did not complete planned SBRT due to ICI-related toxicities, but in the CRC cohort of those who completed protocol treatment, the disease control rate was 37%, and a CRC patient did have an abscopal response.

##### IR + DDRi + ICI

One of the few trials to combine IR+DDRi+ICI was a phase 1 dose-escalation study of peposertib (DNA-PKi) for advanced/metastatic solid tumors combined with avelumab (aPD-L1) without RT (part A) or with palliative XRT (part B). XRT of 30 Gy/10 fx to 3 or fewer sites of disease was delivered concurrently with peposertib, which was then discontinued after the course of radiotherapy in part B. No objective responses were reported and the MTD of peposertib with avelumab and palliative XRT was 250 mg QD. This trial only contained a relatively small cohort of CRC patients (N = 6), making interpretation difficult [64]. As the toxicity of any IR modality combined with a DDRi +/- ICI heavily depends on the anatomical site(s) being treated, this highlights the importance of careful consideration of the best timing of combination therapy regimens.

## 4. Discussion

Despite compelling preclinical evidence and rationale, clinical trials integrating particle radiation with ICI and/or DDRi remain sparse. This likely reflects limited access to particle radiation facilities, the cost of radiation and ICIs, and concerns of increased normal tissue toxicity with DDRi. Additional barriers include identifying the optimal dose/fractionation of the radiotherapy regimen, sequencing and dosing of DDRi/ICI, and the risks and complexities associated with combining multiple novel therapies.

Clinically, particle radiation monotherapy shows promise in LRRC and oligometastatic settings, delivering robust local control with a favorable toxicity profile; however, distant relapses underscore the need for management of systemic disease. Combinations of XRT and DDRi have been few, with notable toxicity when irradiating large elective fields of uninvolved normal GI mucosa. As particle therapy has excellent sparing of normal tissue compared to XRT, it may be ideal for combination therapy with DDRi, but only in contexts where the radiotherapy field is constrained to gross disease (e.g., SBRT of oligometastatic disease, locoregionally recurrent re-irradiation, or as a cone-down boost).

Combinations of XRT + ICI from the bulk of the clinical data on radiation with ICI have compared favorably to historical results with conventional therapies for pMMR disease, but have not approached the striking efficacy of ICI monotherapy for dMMR disease. The addition of a DDRi to XRT might enhance its immune-priming effect to improve the responses to ICI in pMMR disease, but may increase the risk of normal tissue toxicity. The use of particle therapy instead of XRT may add additional immunostimulation and reduce toxicity risks owing to superior normal tissue sparing. With enhanced local control from high-LET particle therapy, doses of DDRi and/or radiation may ameliorate toxicity risks while still achieving local control and systemic anti-tumor immunity. Collectively, these factors underscore the importance of careful trial design for combination therapy.

Future research should focus on refining the dose, fractionation, timing, and order of combinations of particle radiation, DDRi, and ICI to maximize systemic anti-tumor immunity and recurrence-free survival with acceptable toxicity. There is also little data on whether specific classes of particle radiation, DDRi, or ICI, offer superior synergy. Investigating the influence of various intrinsic germline DDR deficiencies beyond MMR on therapeutic outcomes will help tailor personalized treatments. Biomarkers predicting the response and toxicity to particle radiation combinations can guide patient selection and improve overall treatment success.

## 5. Conclusions

Preclinical data support the immunostimulatory effects of high-LET particles and DDR inhibition, demonstrating enhanced ICD, TME remodeling, and antitumor immunity induction, leading to superior control and abscopal effects in preclinical studies (discussed in the companion manuscript). However, clinical data from trials using combinations of particle radiation with DDRi and/or ICI are currently extremely limited. At present, the strongest clinical support for particle therapy lies in achieving local control while sparing the normal tissue. The literature on XRT + ICI is promising compared to standard therapies, but there is room for significant improvement in efficacy. The combination of DDRi with XRT highlights the need to carefully consider radiation volumes when using potent radiosensitizing agents. These concerns may be addressed using concurrent DDRi only when treating gross disease, using DDRi classes with more favorable therapeutic windows, or using particle radiation for superior normal tissue sparing. If such strategies can be safely integrated into contemporary total neoadjuvant therapy frameworks, they may increase complete response rates, expand eligibility for non-operative management in appropriately selected patients, and improve outcomes in metastatic and recurrent settings.

## Figures and Tables

**Table 1:** PBT for Locally Advanced Rectal Cancer (LARC).

Study	Design, Disease (N)	Dose (GyE/fx)	Other Treatments/Arms (%)	Outcomes (% for Rates)	Toxicity (% G3+)
PRORECT trial NCT04525989 [23]	Phase 2, MC, primary LARC pts randomized to PBT or XRT short course RT, RAPIDO inclusion criteria (N = 20 total, 10 PBT and 10 XRT)	25 Gy/5 fx	Randomized to PBT or XRT short course →CAPOX x4 →surgery or WW	pCR: 20 PBT; 10 XRT	Surgical Complications (Clavien–Dindo). Early: CD3a: PBT 30; XRT 20. CD3b: PBT 20; XRT 10
[24]	Retrospective of LCCRT→delayed Surgery vs. SC→chemo→surgery. LCCRT was all XRT, but SC were treated with PBT or XRT (N = 122 total, 18 with PBT, 14.8%)	25 Gy/5 fx	RT⟶Chemo [FOLFOX, XELOX, TEGAFOX, 5FU, tegafur-uracil, FOLFIRI, cape]⟶surgery +- adj ch	pCR/npCR: ≥16 wk RT-surgery Interval: 30/40; <16 wk: 0/25	Unspecified

Abbreviations: CAPOX, capecitabine plus oxaliplatin; CD, Clavien–Dindo classification; CEA, carcinoembryonic antigen; fx, fractions; G3+, grade ≥3 toxicity; GyE, gray equivalent; LARC, locally advanced rectal cancer; MC, multicenter; npCR, near-pathologic complete response; pCR, pathologic complete response; PBT, proton beam therapy; RT, radiotherapy; SC, short-course radiotherapy; WW, watch-and-wait; XRT, X-ray radiotherapy. Studies did not report the MMR/MSS status, unless otherwise noted.

**Table 2:** PBT for Locoregionally Recurrent Rectal Cancer (LRRC).

Study	Design, Disease (N)	Dose (GyE/fx)	Other Treatments/Arms (%)	Outcomes (% for Rates)	Toxicity (% G3+)
[21]	Prospective, LRRC (N = 7 pelvic recurrences)	Varied, all 1.8 Gy/fx PBT plans with IMRT comparisons	86 concurrent ch [5FU, Xeloda], 29 surgery	PBT improved OAR sparing over XRT plans. Mortality: 43. Local failure 43. Distant metastasis: 14. M-FU: 14mo. CR = 1/7. PR = 3/7	Early: G3: GI 43. Late: G4: GI 29
[25]	Retrospective SC, LRRC (N = 6 rectal)	39–45 Gy total at 1.5 Gy/fx delivered BID	100 concurrent ch [5FU or cape], no surgery	OS 1Y/2Y/M: 68/68/39mo. PFS 1Y/2Y/M: 59/47/15mo	Early: G3: lymphopenia 17. Late: 0
[26]	Retrospective, LRRC (N = 28)	Variable, median re-RT dose of 44.4 Gy with 75% treated BID	Neo: 29 Systemic, 7 surgery. 86 concurrent ch. Adj: 21 Surgery	OS 1Y/M: 82/29mo. LC 1Y/M: 66/23mo. PFS 1Y/M: 45/12mo	Early: 11; G3: GI 7, skin 4. Late: 14; G3: GI 7, infection 4; G5: GI 4 (intra-abdominal hemorrhage)
[27]	Retrospective SC, LRRC (N = 13)	Variable patterns of 50–79.2 Gy/18–38 fx (BED10 of 62.5–105.3, 4 fx per week, +/− concurrent ch)	46 concurrent ch [S-1]	OS 3Y/M: 71/67mo. LC 3Y: 80. PFS 3Y: 12	0
[28]	Retrospective, LRRC without prior RT (N = 23)	Variable, 60–87 Gy/25–35 fx QD	4 concurrent ch [Folfox+Bev], 4 concHyperT	OS 3Y/5Y/M: 72/45/54mo. LC 3Y/5Y: 55/47. PFS 3Y/5Y: 38/38. CR = 1/23. PR = 4/23	Early: 0. Late: G4: GI 13
[29]	Retrospective, LRRC with prior RT (N = 10)	Variable, 56–77 Gy/24–37 fx QD	20 concurrent ch [iri+S-1, S-1]	OS 1Y/2Y/M: 100/60/26mo. LC 1Y/2Y: 70/58. PFS 1Y/2Y: 20/10	Early: 0. Late: G4: GI 10

Abbreviations: BID, twice daily; CR, complete response; fx, fractions; FU, follow-up; G3+, grade ≥3 toxicity; GI, gastrointestinal; Gy, gray; LC, local control; LRRC, locoregionally recurrent rectal cancer; OS, overall survival; PBT, proton beam therapy; PFS, progression-free survival; PR, partial response. Studies did not report the MMR/MSS status, unless otherwise noted.

**Table 3:** CIRT for LRRC.

Study	Design, Disease (N)	Dose (GyE/fx)	Other Treatments/Arms (%)	Outcomes (% for Rates)	Toxicity (% G3+)
PANDORA-01 NCT01528683 [30,31]	Phase 1/2 single arm study of CIRT at escalating doses for LRRCSC, LRRC (N = 19)	36–51 Gy/12–17 fx (all at 3 Gy/fx)	No surgery	Mortality: 16%. Local failure: 21%. Distant metastasis: 16%. M-FU: 8mo	0
[32,48]	Phase 1/2, SC, study of escalating CIRT doses for LRRC with no prior RT (N = 235. 2016: N = 180, phase 1/2: 37/phase 2: 143)	67.2–73.6 Gy/16 fx, 4 fx per week	No surgery, no ch < 4 wk	OS 3Y/5Y: 67/46. LC 3Y/5Y: 90/88. FORMERLY (2016): OS 3Y/5Y: 67.2 Gy: 20/20; 70.4 GyE: 52/26; 73.6 Gy: 78/59 (phase 1/2 + 2). LC 5Y (competing risks): 67.2 GyE: 80; 70.4 Gy: 90; 73.6 Gy: 95. *p* = 0.02 (phases 1/2 + 2). CR = 20/186. PR = 59/186 (lesion)	Early: G3: 0.4 GI. Late: G3: 1 skin, 0.4 GI 2016: Phase 1: 0. Phase 2: Late: G3: skin 1, GI 1
[33]	Retrospective (but included 143 pts from [32]) MC, LRRC (N = 224). Excluded for recurrent tumor abutting bowel/bladder, active infection. Spacers used	70.4 or 73.6 Gy/16 fx, 4 fx per week	Ch allowed prior to or after CIRT. 4 pts had prior RT history	OS 3Y/5Y: 73/51. LC 3Y/5Y: 93/88. RC 3Y/5Y: 63/49. PFS 3Y/5Y: 40/27	Early: G3: GI 0.4, infection 1. Late: G3: skin 1, GI 1, infection 3, neuropathy 0.4
UMIN000014513 [49]	Phase 2, SC, CIRT for any isolated recurrent tumor with prior irradiation, excluded for bowel or blood vessel invasion, active infection, or recurrence in <3 months (N = 22 total including other disease sites, 5 were LRRC)	57.6–73.6 Gy/12–16 fx	No concurrent treatment	OS 2Y: 100, LC 2Y: 50 (rectal)	Early: 0 Late: G3: GU 0–50
[34]	Retrospective SC, unresectable LRRC (N = 25). 17 pts had prior RT	48–75.6 Gy/16–21 fx, 5 fx per week. 7 pts got concurrent cape	28 neo ch, 12 adj ch, 4 adj surgery [5FU based]	OS 1Y/2Y: 83/65. LC 1Y/2Y: 90/72. cPR 6/25, cCR 1/25 pCR 2/25	Early: 0 Late: G3: GI 4, neuropathy 4, infection 4
[35]	Retrospective SC, unresectable with prior RT, LRRC (N = 14)	35–76.8 Gy/15–20 fx	0 surgery	OS 1Y/2Y: 100/76. LC 1Y/2Y: 78/52. DMFS 1Y/2Y/M: 64/43/14mo	0
[36]	Retrospective SC, LRRC with prior RT history. Recurrent tumor > 3 mm from luminal organs (N = 77)	70.4 Gy/16 fx, 4 days per week	No surgery, no ch < 4 wk prior to CIRT	OS 3Y/5Y: 61/38/47mo. LC 3Y/5Y: 69/62. RC 3Y/5Y: 85/81. PFS 3Y/5Y: 33/25	Early: G3: infection 6, general 3, neuropathy 1. Late: G3: infection 17, GI 12, skin 1, general 3, neuropathy 5
[37]	Retrospective SC, LRRC after pre-operative RT for initial treatment (N = 7)	57.6–73.6 Gy/12–16 fx, 4 fx per week	Neo: 43 ch [FOLFOX, FOLFIRI], 29 Bev. Adj: 57 ch,43 Bev, 14 XRT [sox, XELOX, cape]	OS 2Y: 100 LC 2Y: 83 PFS 2Y: 29	Early: 0 Late: G3: GI 14, GU 14
[38]	Retrospective SC, unresectable LRRC with prior RT (N = 24) No invasion of GI tract or bladder allowed	67.5 Gy/15 fx, (45.8%) 72 Gy/20 fx (41.7%) or 75.6 Gy/21 fx (12.5%)	Ch: 1 neo, adj 29, both 42. 0 surgery	OS 1Y/2Y/M: 87/81/41mo. LC 1Y/2Y: 100/93. PFS 1Y/2Y: 71/45. CR = 1/24. PR = 4/24	Early: 0. Late: G3: GI 4, skin 4, infection 4
GUNMA0801 [39,40]	Phase 2, SC, LRRC no prior RT, no direct invasion of GI tract or bladder (N = 28)	73.6 Gy/16 fx, 4 fx per week	No surgery, no ch < 4 wks prior to CIRT or adj	OS 3Y/5Y/M: 89/50/76mo. LC 3Y/5Y: 88/83. PFS 3Y/5Y/M: 30/23/12mo	Early: 0. Late: G3: infection 7
[41]	Retrospective SC, LRRC for pts without prior RT (nRT, N = 390) vs. with prior RT history (reRT, N = 83). Electivd nodal coverage was allowed for nRT only	nRT 73.6 Gy/16 fx. reRT 70.4 Gy/16 fx, 4 fx per week	Neo: 71 Ch, 18 surgery. No concurrent ch. Spacer placement prior to CIRT or resection of involved bowel (1–2 months after CIRT) allowed	OS 3Y/5Y: nRT: 73/50; reRT: 76/50 LC 3Y/5Y: nRT: 80/72; reRT: 80/69 PC 3Y/5Y: nRT: 54/40/; reRT: 41/36 All 5Y differences ns, OS, and LC no difference ch v no ch	Early: nRT: 1; G3: GI 1. reRT: 7; infection 6, neuropathy 1. Late: nRT: 6; G3: skin 1, 0.3 GU, GI 1, infection 2, neuropathy 1; G4: GI 0.3, infection 1. reRT: 27; G3: skin 2, GI 7, infection 11, neuropathy 6; G4: infection 2. reRT higher G3+ incidence (*p* < 0.05)
[42]	Prospective feasibility SC, unresectable LRRC with or without prior RT with recurrence < 3 mm from bowel (N = 12)	nRT 73.6 Gy/16 fx reRT 70.4 Gy/16 fx, 4 fx per week	CIRT followed by planned resection of the normal bowel <3 mm from recurrent disease (LAR or small bowel resection)	OS 3Y: 90. RFS 3Y: 57; IF:90, OOF: 72. 12/12 ypT0	Early: 0. Late: CIRT: G3: 8 neuropathy. Surgery: CD3a: 8
[43]	Retrospective MC, previously irradiated LRRC comparing CIRT alone at one institution vs. Combined Modality Therapy (CMT) of XRT re-irradiation, immediate resection+ intraoperative electron radiotherapy (IOERT) boost (N = 85 CIRT/86 CMT)	CIRT 70.4 Gy/16fx, 4 fx per week CMT 30 Gy/15 fx. XRT + IOERT 12.5–15 Gy	-CIRT w/o concurrent ch. -Neo ch, RT, immediate surgery + IOERT	OS 2Y/5Y/M: CIRT 83/47/4.5Y. HR vs. CMT 0.5, *p* < 0.01 PC 2Y/5Y/M: CIRT 58/46/3.6Y. HR vs. CMT 1.4, ns DMFS 2Y/5Y/M: CIRT 46/38/1.8Y. HR vs. CMT 1.2, ns PC 2Y/5Y/M: CIRT 31/23/1.1Y. HR vs. CMT 1.3, ns	CIRT: G3–4: GI 13. Early G3–4 (OR CMT vs. CIRT, *p* < 0.05): GI: 22, GU 3, skin 3 Late G3–4 (HR CMT vs. CIRT, *p* < 0.05): GU 33
[44]	Retrospective MC, LRRC with prior RT (N = 35 CIRT/31 XRT) CIRT and XRT performed at different institutions	CIRT 70.4 Gy/16 fx XRT: 50 Gy (range 25–62.5 Gy) with a median of 25 fx (range 3–33)	-CIRT: 40 neo/adj ch -XRT ± concurrent ch (68%)/surgery (36%)	OS 1Y/3Y: CIRT 97/8. HR CIRT vs. XRT 0.30, *p* = 0.004 LC 1Y/3Y: CIRT 94/87. HR CIRT vs. XRT 0.17, *p* = 0.002	Early: G2+: GI 3, GU 9. Late: CIRT: G3+: GI 6. GU+GI adjusted HR (CIRT vs. XRT, *p* < 0.05): 0.15. Skin G1+: 11
[46]	Retrospective MC, LRRC mostly comparing surgery vs. particle RT, not much data presented on the CIRT/PBT (N = 14 PBT/CIRT)	Unspecified	CIRT/PBT, surgery, chRT, Palliative care	OS 3Y/5Y/M: 76/44/47mo. DSS 3Y/5Y: 76/56 OS, DSS similar to surgery, CIRT/PBT better than palliative care	Unspecified
[47]	Retrospective SC, Pelvic recurrence of CRC (N = 114 CIRT, 33 PBT, N = 147 total rectal)	PBT: 60–75 Gy/18–35 fx CIRT: 57.6–73.6 Gy/12–16 fx	No	OS 2Y/3Y: CIRT 94/88; PBT 87/63. LC 1Y/2Y/3Y: (both CIRT and PBT, not reported separately) 91/81/76	Early: 0. Late: CIRT 4, PBT 9. G3: neuropathy 2, infection 1, GI 1, skin 1; G4: GI 1

Abbreviations: CIRT, carbon-ion radiation therapy; CR, complete response; DMFS, distant metastasis-free survival; fx, fractions; G3+, grade ≥3 toxicity; GI, gastrointestinal; GU, genitourinary; GyE, gray equivalent; LC, local control; LRRC, locoregionally recurrent rectal cancer; MC, multicenter; nRT, no prior radiotherapy; OS, overall survival; PFS, progression-free survival; PR, partial response; reRT, re-irradiation; RT, radiotherapy; SC, single-center. Studies did not report the MMR/MSS status, unless otherwise noted.

**Table 4:** PBT/CIRT for oligometastatic disease.

	Study	Design, Disease (N)	Dose (GyE/fx)	Other Treatments/Arms (%)	Outcomes
**PBT**	NCT01239381 [50]	Phase II, SC. PBT SBRT for 1–4 liver metastases L-OM (N = 34 CRC)	30–50 Gy/5 fx	Not excluded	No G3 tox. LC 1yr: 58.8% (CRC)
	[51]	Phase I, SC. Dose escalation for PBT SBRT for1–3 liver metastases (N = 5 CRC, 2 rectal)	36, 48, 60 Gy/3 fx	No other arms	No G3+ tox
	NCT04456621 [52]	Phase II, SC. PBT SBRT1–4 liver metastases. (N = 48 total pts, 30 CRC)	60 Gy/5 fx	8 ch < 6mo	No G3 tox. OS 6mo: 90. LC 6mo/1Y: 100/89 (CRC). ch vs. no ch (1Y): 94 vs. 76, *p* ns. (overall). PFS 6mo: Intrahepatic: 63; Extrahepatic: 59
	[53]	Retrospective, SC. PBT with conventional, hypofractionated, or SBRT of 1–3 liver oligometastases (N = 63 lesions treated, 41 CRC pts, 12 pts rectal)	74–76 Gy/37–38 fx if adjacent to OARs, 72.6 Gy/ 22 fx in hilar region, 64 Gy/8 fx if away from hilum/OARs	Prior ch: 30. Concurrent: 3	No G3 tox. 2yrLC for conventional, hypofractionated, and SBRT of 35, 43.9, and 71.1%, respectively. Median LC times for conventional, hypofractionated, and SBRT of 16.4, 21.4, and 47.3 months, respectively
	[57]	Retrospective, MC. PBT SBRT 1–3 lung oligometastases, (N = 118 total pts, 50 CRC, 27 rectal)	64 Gy/10 fx	33 neo ch, 18 adj ch, 1 surgery	1 G3 dermatitis. Local progression-free survival 1.2 years: 72.7 and 65.8%. CRC primary identified as poor prognostic indicator of LC
**CIRT**	UMIN000032911 [54]	Phase I, SC. Dose escalation of single fx CIRT for unresectable liver metastases >5 mm from bowel with no other sites of disease. CRC (N = 31 CRC pts, 14 rectal)	Escalated from 36 to 58 Gy/1 fx.	No surgery, no ch < 4 wk	No acute G3+ tox, 2/8 pts at 53 Gy dose level with hilar disease had late biliary obstruction. 3yrLC was 82% for 53 and 58 Gy dose levels, and 28% for lower doses
	[55]	Retrospective, MC. CIRT for liver oligometastatic disease from any site. (N = 102 total pts, 60 CRC)	Variable, most commonly 60 Gy/12 fx (68/121 lesions)	No surgery	No acute G3+ tox. For the CRC cohort, 1 and 2yrLC of 86.5 and 73.8%, respectively
	[56]	Retrospective, SC. SBRT or hypofractionated CIRT for CRC liver or lung metastases. CRC (N = 19 CRC pts, 8 rectal, 23 lesions treated)	60 Gy/4 fx 60 Gy/12 fx (if close to bowel) 64.8 Gy/12 fx (if tumor > 5 cm)	42 neo ch, 11 adj ch	No acute G3+ tox. 2yrLC 67% for all lesions; 83% for lung (all 9 received 60 Gy/4 fx) and 61% for liver (4/14 pts had local failure in the liver; 2/4 pts after 64.8 Gy/12 fx and 2/9 pts after 60 Gy/4 fx)
	[58]	Retrospective, SC. CIRT SBRT for 1–4 pulmonary metastases. (N = 34 CRC pts, 19 rectal. 44 lesions)	Various but >70% received 60 Gy/4 fx	No surgery, no ch < 1mo	No G3+ tox. 3yrLC rate of 85.4%
	[59]	Retrospective, MC. Hypofractionated CIRT for any disease site with LN oligorecurrence. (N = 323 pts total, 77 CRC)	Variable, most commonly 48–52.8 Gy/12 fx, 4 fx per week	Not excluded	2yrLC 80.6% for CRC pts. No acute G3+ tox for CRC pts but 2/77 had G2 duodenitis
	[45]	Retrospective, SC. Hypofractionated CIRT for isolated PA lymph node recurrence. (N = 34 total, 20 rectal)	Variable, but >85% received 52.8 Gy/12 fx, 4 fx per week	29 adj ch, 41 neo ch	No G3+ tox. 2, 3, and 5yrLC 70.1%. Complete response, partial response, and stable disease rates of 38.2, 17.6, and 26.5%. Of the pts that passed away, 12/13 died of distant failure.
**CIRT vs. PBT and/or XRT**	[60]	Retrospective, MC. Oligorecurrence of CRC in only the PA lymph nodes comparing CIRT to XRT (N = 116, 63 XRT, 53 CIRT. 45% rectal)	CIRT: 48–55.2 Gy/12 fx, 4 fx per week. No concurrent ch, possible elective coverage. XRT: Variable, 22/63 pts had conventional fractionation, 39/63 hypofractionated, and 2 pts had SBRT. 6/63 had concurrent ch	CIRT (40 neo ch, 4 adj ch [FOLFOX, FOLFIRI, CAPOX, etc]) vs. XRT (70 ch)	3.2% G3 tox (diarrhea) in XRT. 2 and 5yrLC for CIRT of 78.9 and 62%, respectively; 63.3 and 37.5% for XRT.
	[61]	Retrospective, MC. CIRT/PBT vs. XRT (conventional or SBRT) for pulmonary/liver oligometastases or PALN recurrence from any primary site (N = 132 total pts, 48 CRC; 85 PBT, 47 CIRT, Arms: Lung: 48, Liver: 102, LN: 43)	Very variable, ranged from single fx to conventional fractionation with potentially more SBRT/hypofractionation in CIRT > PBT > XRT	Not excluded	G3 tox < 3.5%. 3yrLC CIRT/PBT: 72.8–83.2% for all sites. For the CRC cohort, the incidence rate ratio of local failure showed improved LC for CIRT/PBT in the liver, with a non-significant trend towards less local failure in lungs and lymph nodes compared to XRT SBRT. Non-significant trend towards improved LC with CIRT compared to PBT in liver
	[62]	Retrospective, MC. CIRT or PBT for 1–3 liver oligometastases from any primary site (N = 322 total, 51.9% CRC. N = 234 PBT, 88 CIRT)	Variable, ranging from 58 Gy/1 fx for CIRT to conventionally fractionated for PBT	14 concurrent systemic (overall) [S-1, etc]	2 and 5yrLC of 74.3 and 66.4% for all primary sites. CIRT and PBT not compared for LC. Trend towards better survival in CRC cohort with single lesions < 5 cm

Abbreviations: CIRT, carbon-ion radiation therapy; CRC, colorectal cancer; fx, fractions; G3+, grade ≥ 3 toxicity; GyE, gray equivalent; LC, local control; LN, lymph node; OM, oligometastatic; OS, overall survival; PA, para-aortic; PBT, proton beam therapy; PFS, progression-free survival; SBRT, stereotactic body radiation therapy. Studies did not report the MMR/MSS status, unless otherwise noted.

**Table 5:** Combinations of IR with DDRi and/or ICI.

	Study, MMR/MSS Status # of Patients, (% Total)	Design, Disease (N)	Dose (GyE/fx)	ICI/DDRi (Timing)	Outcomes & Toxicity
PBT or XRT +DDRi	NCT01589419 [63] MSS = 32 (100%)	Phase 1b dose-escalation study of the PARP inhibitor veliparib concurrent with LCCRT in LARC followed by surgery (N = 32)	50.4 Gy/28 fx	Concurrent with LCCRT	pCR rate of 29%. 9% G3 diarrhea
XRT+DDRi+ICI	NCT03724890 [64] MSS NR	Phase 1 dose-escalation study of peposertib (DNA-PKi) for advanced/metastatic solid tumors (N = 6 CRC, 1 rectal). Avelumab (aPD-L1) without (part A) or with palliative XRT (part B) RT was to ≤ 3 sites	30 Gy/10 fx	Avelumab (q2w) + Peposertib (BID or QD). In part B, peposertib was given concurrently with RT but not continued afterwards	MTD for peposertib with avelumab and RT was 250 mg QD. No objective responses were observed. G3–4 tox: DDRi 0–17%, ICI 0–67%, and 0–17% attributed to XRT
PBT/XRT+ICI	NCT03104439 [65] MSS = 40 (100%)	Phase 2 single arm, Metastatic MSS CRC treated with cycles of Ipi (q2w)/Nivo(q6w) (aCTLA4/aPD-1) and SBRT (CRC N = 40, 2 rectal; 27 received SBRT). XRT and PBT not compared	24 Gy/3 fx with cycle #2 of Ipi/nivo	Ipi/Nivo → Nivo → SBRT+Ipi/Nivo	Intention to treat disease control rate (DCR): 25%. Per-protocol DCR: 37%. Ipi/Nivo toxicity: G3 53%, G4 15%, G5 3%. RT toxicity: G4 15%; G4 7%
XRT+ICI	TORCH NCT04518280 [66] MSS = 121 (100%)	LARC who were treated with either short course RT (SCRT) followed by CAPEOX x6 cycles with toripalimab (aPD-1) (group A, N = 62) or the same regimen, but SCRT was delivered in between cycles 2 and 3 of CAPEOX+toripalimab (group B, N = 59)	25 Gy/5 fx	Concurrent with CAPOX	54–57% CR in both arms (with CR including cCR + pCR of pts that went to surgery). G3–G4 acute adverse effects 45% in group A, 42% group B
XRT+ICI	PRIME RT NCT04621370 [67,68] MSS = 39 (93%)	Phase II randomized trial of SCRT (Arm A, N = 21) or LCCRT (Arm B, N = 21) followed by FOLFOX with concurrent durvalumab (aPD-L1) during both RT and ch	25 Gy/5 fx or 50 Gy/25 fx	Durvalumab (aPD-L1) concurrent with both RT and ch	6-months combined cCR+pCR rate of 67% (SCRT, arm A) vs. 48% (LCCRT, arm B). 1-year post-treatment pCR+sustained cCR rates of 61 vs. 38%, respectively 10% G3 tox per arm
XRT+ICI	UNION NCT04928807 [69] MSS = 231 (100%)	PHASE III randomized trial of SCRT followed by CAPEOX+camrelizumab (aPD-1) (N = 113) or LCCRT followed by CAPEOX x2 cycles (N = 118). All pts underwent surgery and adj systemic therapy	25 Gy/5 fx or 50.4 Gy/28 fx	Concurrent with CAPEOX in the SCRT arm only	pCR rate of 39.8% in the SCRT → CAPEOX+camrelizumab arm vs. 15.3% in the LCCRT → CAPEOX arm. G3+ tox was 29.2% and 27.2%, respectively
XRT+ICI	STELLAR II NCT05484024 [70] MSS = 218 (100%)	Phase 2 RCT of SCRT followed by CAPEOX x4 or FOLFOX x6 with (iTNT, N = 110) or without (TNT, N = 108) sintilimab (aPD-1), followed by TME or NOM depending on cCR	25 Gy/5 fx	Concurrent with CAPEOX or FOLFOX for 4 cycles. Only 1/204 pts received FOLFOX, all others received CAPEOX	CR (cCR+pCR) rates of 45.5 vs. 25.0% after iTNT and TNT cohorts respectively (*p* = 0.003). G3–4 treatment-related AE rates of 34.5% of the iTNT group vs. 19.4% of the TNT group
XRT+ICI	SPRING-01 ChiCTR2100052288 [71] MSS = 84 (86%)	Randomized phase 2 trial of LARC pts treated with SCRT followed by CAPEOX x6 with or without sintilimab (aPD-1) followed by surgery for all pts. 84–88% were pMMR, 2% dMMR and 10–14% unknown status (N = 98, 49 each arm)	25 Gy/5 fx	Concurrent with CAPEOX	CR (cCR+pCR) rate of 61.2% with sintilimab vs. 32.7% with CAPEOX alone (*p* = 0.009). G3–4 treatment-related AE of 33% in both arms. G5 rate of 2% in the CAPEOX alone arm
XRT+ICI	AVERECTAL NCT03503630 [72] MSS NR	Single arm phase 2 trial of SCRT followed by FOLFOX+avelumab (aPD-L1) x6 followed by surgery (N = 44)	25 Gy/5 fx	Concurrent with FOLFOX	25% with pCR and an additional 25% with near-pCR. G3, G4 and G5 treatment-related AE rates of 58.1%, 11.6% and 2.3%, respectively. 35% related to TME, 0% related to avelumab
XRT+ICI	PRECAM NCT05216653 [73] MSS = 34 (100%)	Single arm phase 2 of MSS LARC treated with SCRT followed by CAPEOX+Envafolimab (aPD-L1) and then TME at 2 wks after chemoimmunotherapy (N = 34)	25 Gy/5 fx	Envafolimab was given weekly concurrent with 2 cycles of CAPEOX	pCR 62.5%. G3 treatment-related AE rate of 6.3%
XRT+ICI	NRG-GI002 NCT02921256 [74] MSS NR	Randomized phase 2 trial comparing FOLFOX x6 followed by LCCRT with (N = 90) or without (N = 95) pembrolizumab (aPD-1) starting the first day of LCCRT and continuing for up to 6 cycles (q3w). Resection was 8–12 wks after LCCRT. Pts were higher risk/potentially more advanced than typical LARC cohorts with inclusion criteria of cN2, cT4 or distal cT3, bulky or <3 mm from MRF, or not a candidate for sphincter-sparing surgery	50.4 Gy/28 fx	Concurrent with and after LCCRT	Similar rates of pCR, cCR, and NAR scores were similar between arms (29–32%, 13.6–13.9%, and score of 11.5–14 out of 100, respectively). G3–4 tox of 48.2% with aPD-1 vs. 37.3% without
XRT+ICI	VOLTAGE NCT02948348 [75] MSS = 39 (89%)	Phase 1 study of LCCRT followed by nivolumab (aPD-1) x5 followed by TME 10–12 wks after the completion of LCCRT (N = 44)	50.4 Gy/28 fx	Nivolumab started within 14 days after completion of LCCRT	In MSS pts, 3yrRFS 79.5%, 3yrOS 97.4%. cCR 20.5%, pCR 28.9%. Serious adverse events in 8 pts, 2 immune-related G3
XRT+ICI	PANDORA NCT04083365 [76] MSS = 46 (84%)	Single arm phase 2 study of LCCRT followed by durvalumab (aPD-L1) x3 and finally surgery 10–12 wks after completion of LCCRT (N = 55)	50.4 Gy/25–28 fx	Durvalumab started 1 week after completion of LCCRT	pCR 34.5%, G3 immune-related AE in 7.3%
XRT+ICI	NCT04340401 [77] pMMR = 25 (100%)	Phase 2 study of CAPEOX+camrelizumab (aPD-1) x3 followed by LCCRT and then an additional 2 cycles of CAPEOX alone (N = 25)	50.6 Gy/22 fx	Concurrent with initial CAPEOX	pCR 33.3%(with an additional 38% with major pathologic response) cCR 48%, G3 tox in 25%, 0% immune-related
XRT+ICI	NECTAR NCT04911517 [78] MSS = 50 (100%)	Single arm phase 2 study of pts treated with LCCRT with capecitabine + tislelizumab (aPD-1) for 3 cycles q3w(2 concurrent and 1 after LCCRT), with TME 6–12 wks after completion of LCCRT (N = 50)	50 Gy/25 fx	2 cycles concurrent with LCCRT and 1 after with capecitabine (q3w)	pCR 40%. 4% G3 treatment-related AE, 2% immune-related
XRT+ICI	NCT04304209 [79] MSS = 134 (100%)	Phase 2 randomized trial examining CAPOX x4 with (N = 67) or without (N = 67) concurrent sintilimab (aPD-1) followed by LCCRT and then surgery or NOM	50 Gy/25 fx	Concurrent with CAPEOX	CR (cCR+pCR) rate of 26.9 and 44.8% in the control and sintilimab arms, respectively G3–4 immune-related AE in aPD-1 were 6–14%, similar toxicity profile in control.
XRT+ICI	POLARSTAR NCT05245474 [80] MSS = 151 (88%)	Phase 2 randomized trial with three arms examining LCCRT followed by surgery with tislelizumab x3 concurrently with LCCRT (group A, N = 59), after LCCRT (group B, N = 55), or not at all (control arm, N-57). Surgery was 6–12 wks after LCCRT for the control groups and 8–12 wks after LCCRT for groups A and B	45–50.4 Gy/25–28 fx	Tislelizumab was started 1 week after initiation of LCCRT (group A) or 2 wks after the end of LCCRT (group B)	pCR rates were 27.1, 32.7, and 14.0% for groups A, B, and control, respectively. G3–4 AEs were 3%, 5%, and 0%, and grade 3–4 surgical complication rates were 5%, 5%, and 4%, respectively

Abbreviations: AE, adverse event; cCR, clinical complete response; CR, complete response; CTLA-4, cytotoxic T-lymphocyte–associated protein 4; DDRi, DNA damage response inhibitor; fx, fractions; G3–4, grade 3–4 toxicity; Gy, gray; ICI, immune checkpoint inhibitor; IR, ionizing radiation; LCCRT, long-course chemoradiotherapy; pCR, pathologic complete response; PD-1, programmed death-1; PD-L1, programmed death-ligand 1; PBT, proton beam therapy; SCRT, short-course radiotherapy; TNT, total neoadjuvant therapy; XRT, X-ray radiotherapy. Studies did not report the MMR/MSS status, unless otherwise noted.

## Data Availability

No new data were created or analyzed in this study. Data sharing is not applicable to this article.

## Supplementary Materials

The following supporting information can be downloaded at: https://www.mdpi.com/article/10.3390/cancers18040652/s1. File S1: The PICO search strategy, specific search strategies, and keywords for pMMR status identification.

## References

[1] Bray F., Laversanne M., Sung H., Ferlay J., Siegel R.L., Soerjomataram I., Jemal A. (2024). Global cancer statistics 2022: GLOBOCAN estimates of incidence and mortality worldwide for 36 cancers in 185 countries. CA Cancer J. Clin..

[2] Du M., Drew D.A., Goncalves M.D., Cao Y., Chan A.T. (2025). Early-onset colorectal cancer as an emerging disease of metabolic dysregulation. Nat. Rev. Endocrinol..

[3] van Geffen E.G.M., Hogewoning C.R.C., Hazen S.J.A., Sluckin T.C., Lange M.M., Snaebjornsson P., Beets-Tan R.G.H., Marijnen C.A.M., Verhoef C., Chalabi M. (2025). Incidence and Outcomes of Patients With Mismatch Repair Deficient Rectal Cancer Operated in 2016: A Nationwide Cohort From The Netherlands. Clin. Color. Cancer.

[4] Williams C.J.M., Peddle A.M., Kasi P.M., Seligmann J.F., Roxburgh C.S., Middleton G.W., Tejpar S. (2024). Neoadjuvant immunotherapy for dMMR and pMMR colorectal cancers: Therapeutic strategies and putative biomarkers of response. Nat. Rev. Clin. Oncol..

[5] NCCN NCCNClinical Practice Guidelines in Oncology (NCCN Guidelines) for Rectal Cancer V.3.2025 NCCN.Org: National Comprehensive Cancer Network. Inc 2025. https://www.nccn.org/guidelines/guidelines-detail?id=1461.

[6] Anker C.J., Tchelebi L.T., Selfridge J.E., Jabbour S.K., Akselrod D., Cataldo P., Abood G., Berlin J., Hallemeier C.L., Jethwa K.R. (2024). Executive Summary of the American Radium Society on Appropriate Use Criteria for Nonoperative Management of Rectal Adenocarcinoma: Systematic Review and Guidelines. Int. J. Radiat. Oncol. Biol. Phys..

[7] Rummel K.A., Sutton E.A., Hallemeier C.L., Merrell K.W., Callaghan C.M., Haddock M.G., Waddle M.R., Jethwa K.R. (2023). Non-Operative Management of Rectal or Anal Canal Adenocarcinoma: National Cancer Database Analysis of the Impact of Disease, Treatment, and Social Determinants of Health on Overall Survival. Int. J. Radiat. Oncol. Biol. Phys..

[8] O’Connor M.J. (2015). Targeting the DNA Damage Response in Cancer. Mol. Cell.

[9] Amodio V., Vitiello P.P., Bardelli A., Germano G. (2024). DNA repair-dependent immunogenic liabilities in colorectal cancer: Opportunities from errors. Br. J. Cancer.

[10] Le D.T., Uram J.N., Wang H., Bartlett B.R., Kemberling H., Eyring A.D., Skora A.D., Luber B.S., Azad N.S., Laheru D. (2015). PD-1 Blockade in Tumors with Mismatch-Repair Deficiency. N. Engl. J. Med..

[11] Cercek A., Bachet J.B., Capdevila J., Starling N., Chen E., Salvatore L., Bando H., O’Donnell S., Harfst L., Szijgyarto Z. (2025). A Phase Two, Single-Arm, Open-Label Study With Dostarlimab Monotherapy in Participants With Untreated Stage II/III dMMR/MSI-H Locally Advanced Rectal Cancer (AZUR-1). Clin. Color. Cancer.

[12] Concannon K., Morris B.B., Gay C.M., Byers L.A. (2023). Combining targeted DNA repair inhibition and immune-oncology approaches for enhanced tumor control. Mol. Cell.

[13] Zhang Q., Jiang L., Wang W., Huber A.K., Valvo V.M., Jungles K.M., Holcomb E.A., Pearson A.N., The S., Wang Z. (2024). Potentiating the radiation-induced type I interferon antitumoral immune response by ATM inhibition in pancreatic cancer. JCI Insight.

[14] Nikitaki Z., Velalopoulou A., Zanni V., Tremi I., Havaki S., Kokkoris M., Gorgoulis V.G., Koumenis C., Georgakilas A.G. (2022). Key biological mechanisms involved in high-LET radiation therapies with a focus on DNA damage and repair. Expert. Rev. Mol. Med..

[15] Zhang J., Si J., Gan L., Zhou R., Guo M., Zhang H. (2021). Harnessing the targeting potential of differential radiobiological effects of photon versus particle radiation for cancer treatment. J. Cell. Physiol..

[16] Zhou Q., Howard M.E., Tu X., Zhu Q., Denbeigh J.M., Remmes N.B., Herman M.G., Beltran C.J., Yuan J., Greipp P.T. (2021). Inhibition of ATM Induces Hypersensitivity to Proton Irradiation by Upregulating Toxic End Joining. Cancer Res..

[17] Huang R.-X., Zhou P.-K. (2020). DNA damage response signaling pathways and targets for radiotherapy sensitization in cancer. Signal Transduct. Target. Ther..

[18] Bright S.J., Flint D.B., Martinus D.K.J., Turner B.X., Manandhar M., Ben Kacem M., McFadden C.H., Yap T.A., Shaitelman S.F., Sawakuchi G.O. (2022). Targeted Inhibition of DNA-PKcs, ATM, ATR, PARP, and Rad51 Modulate Response to X Rays and Protons. Radiat. Res..

[19] Du J., Kageyama S.I., Hirata H., Motegi A., Nakamura M., Hirano Y., Okumura M., Yamashita R., Tsuchihara K., Hojo H. (2021). Comparative analysis of the immune responses in cancer cells irradiated with X-ray, proton and carbon-ion beams. Biochem. Biophys. Res. Commun..

[20] Jeans E.B., Jethwa K.R., Harmsen W.S., Neben-Wittich M., Ashman J.B., Merrell K.W., Giffey B., Ito S., Kazemba B., Beltran C. (2020). Clinical Implementation of Preoperative Short-Course Pencil Beam Scanning Proton Therapy for Patients With Rectal Cancer. Adv. Radiat. Oncol..

[21] Berman A.T., Both S., Sharkoski T., Goldrath K., Tochner Z., Apisarnthanarax S., Metz J.M., Plastaras J.P. (2014). Proton Reirradiation of Recurrent Rectal Cancer: Dosimetric Comparison, Toxicities, and Preliminary Outcomes. Int. J. Part. Ther..

[22] Ma J., Dragojevic S., Remmes N., Mendelson N., Kloeber J., Ebner D., Wu Z., Gunn H., Merrell K., Hallemeier C. (2025). Linear energy transfer optimized proton therapy for rectal cancer. Radiother. Oncol..

[23] Valdman A., Marsk R., Karimi M., Asklid D., Brattstrom D., Ostling Palme J., Martling A., Nilsson P.J. (2024). Surgical outcomes following total neoadjuvant therapy in rectal cancer with short-course radiotherapy using protons or photons: Initial safety data from the PRORECT randomized trial. Br. J. Surg..

[24] Lin Y.H., Hsu H.C., Huang E.Y. (2025). Prognostic Value of Pretreatment Carcinoembryonic Antigen (CEA) in Rectal Cancer Treated with Preoperative Short-Course Radiotherapy with Delayed Surgery or Long-Course Radiotherapy. Onco Targets Ther..

[25] Moningi S., Ludmir E.B., Polamraju P., Williamson T., Melkun M.M., Herman J.D., Krishnan S., Koay E.J., Koong A.C., Minsky B.D. (2019). Definitive hyperfractionated, accelerated proton reirradiation for patients with pelvic malignancies. Clin. Transl. Radiat. Oncol..

[26] Koroulakis A., Molitoris J., Kaiser A., Hanna N., Bafford A., Jiang Y., Bentzen S., Regine W.F. (2021). Reirradiation for Rectal Cancer Using Pencil Beam Scanning Proton Therapy: A Single Institutional Experience. Adv. Radiat. Oncol..

[27] Hiroshima Y., Ishikawa H., Murakami M., Nakamura M., Shimizu S., Enomoto T., Oda T., Mizumoto M., Nakai K., Okumura T. (2021). Proton Beam Therapy for Local Recurrence of Rectal Cancer. Anticancer. Res..

[28] Takagawa Y., Suzuki M., Yamaguchi H., Seto I., Azami Y., Machida M., Takayama K., Tominaga T., Murakami M. (2023). Outcomes and Prognostic Factors for Locally Recurrent Rectal Cancer Treated With Proton Beam Therapy. Adv. Radiat. Oncol..

[29] Takagawa Y., Suzuki M., Seto I., Azami Y., Machida M., Takayama K., Sulaiman N.S., Nakasato T., Kikuchi Y., Murakami M. (2024). Proton beam reirradiation for locally recurrent rectal cancer patients with prior pelvic irradiation. J. Radiat. Res..

[30] Habermehl D., Wagner M., Ellerbrock M., Buchler M.W., Jakel O., Debus J., Combs S.E. (2015). Reirradiation Using Carbon Ions in Patients with Locally Recurrent Rectal Cancer at HIT: First Results. Ann. Surg. Oncol..

[31] Combs S., Kieser M., Habermehl D., Weitz J., Jäger D., Fossati P., Orrechia R., Engenhart-Cabillic R., Pötter R., Dosanjh M. (2012). Phase I/II trial evaluating carbon ion radiotherapy for the treatment of recurrent rectal cancer: The PANDORA-01 trial. BMC Cancer.

[32] Yamada S., Kamada T., Ebner D.K., Shinoto M., Terashima K., Isozaki Y., Yasuda S., Makishima H., Tsuji H., Tsujii H. (2016). Carbon-Ion Radiation Therapy for Pelvic Recurrence of Rectal Cancer. Int. J. Radiat. Oncol. Biol. Phys..

[33] Shinoto M., Yamada S., Okamoto M., Shioyama Y., Ohno T., Nakano T., Nemoto K., Isozaki Y., Kawashiro S., Tsuji H. (2019). Carbon-ion radiotherapy for locally recurrent rectal cancer: Japan Carbon-ion Radiation Oncology Study Group (J-CROS) Study 1404 Rectum. Radiother. Oncol..

[34] Cai X., Du Y.Y., Wang Z., Li P., Yu Z., Zhang Q., Zhang Z. (2020). The role of carbon ion radiotherapy for unresectable locally recurrent rectal cancer: A single institutional experience. Radiat. Oncol..

[35] Barcellini A., Vitolo V., Cobianchi L., Peloso A., Vanoli A., Mirandola A., Facoetti A., Fiore M.R., Iannalfi A., Vischioni B. (2020). Re-irradiation With Carbon Ion Radiotherapy for Pelvic Rectal Cancer Recurrences in Patients Previously Irradiated to the Pelvis. In Vivo.

[36] Yamada S., Takiyama H., Isozaki Y., Shinoto M., Ebner D.K., Koto M., Tsuji H., Miyauchi H., Sekimoto M., Ueno H. (2022). Carbon Ion Radiotherapy for Locally Recurrent Rectal Cancer of Patients with Prior Pelvic Irradiation. Ann. Surg. Oncol..

[37] Shiba S., Okamoto M., Shibuya K., Okazaki S., Kobayashi D., Miyasaka Y., Ohno T. (2022). Safety and Efficacy of Re-irradiation With Carbon-ion Radiotherapy for Pelvic Recurrence of Rectal Cancer After Preoperative Chemoradiotherapy: A Retrospective Analysis. In Vivo.

[38] Cai X., Li P., Zhao J.F., Wang W.W., Cheng J.Y., Zhang G.Y., Cai S.J., Zhang Z., Jiang G.L., Zhang Q. (2023). Definitive carbon ion re-irradiation with pencil beam scanning in the treatment of unresectable locally recurrent rectal cancer. J. Radiat. Res..

[39] Shiba S., Okamoto M., Shibuya K., Kobayashi D., Miyasaka Y., Ohno T. (2024). Five-year outcomes in carbon-ion radiotherapy for postoperative pelvic recurrence of rectal cancer: A prospective clinical trial (GUNMA 0801). Clin. Transl. Radiat Oncol..

[40] Shiba S., Okamoto M., Kiyohara H., Ohno T., Kaminuma T., Asao T., Ojima H., Shirabe K., Kuwano H., Nakano T. (2019). Prospective Observational Study of High-Dose Carbon-Ion Radiotherapy for Pelvic Recurrence of Rectal Cancer (GUNMA 0801). Front. Oncol..

[41] Takiyama H., Yamada S., Isozaki T., Ikawa H., Shinoto M., Imai R., Koto M. (2024). Carbon-Ion Radiation Therapy for Unresectable Locally Recurrent Colorectal Cancer: A Promising Curative Treatment for Both Radiation Therapy: Naive Cases and Reirradiation Cases. Int. J. Radiat. Oncol. Biol. Phys..

[42] Kimura K., Takiyama H., Yamada S., Ito K., Koba M., Imada A., Song J., Kataoka K., Kihara T., Matsuda I. (2025). A Novel Treatment Strategy for Unresectable Locally Recurrent Rectal Cancer-Upfront Carbon-Ion Radiotherapy Followed by Surgical Resection of the Irradiated Intestines. Cancers.

[43] Jeans E.B., Ebner D.K., Takiyama H., Qualls K., Cunningham D.A., Waddle M.R., Jethwa K.R., Harmsen W.S., Hubbard J.M., Dozois E.J. (2023). Comparing Oncologic Outcomes and Toxicity for Combined Modality Therapy vs. Carbon-Ion Radiotherapy for Previously Irradiated Locally Recurrent Rectal Cancer. Cancers.

[44] Chung S.Y., Takiyama H., Kang J.H., Chang J.S., Min B.S., Tsuji H., Yamada S., Koom W.S. (2022). Comparison of clinical outcomes between carbon ion radiotherapy and X-ray radiotherapy for reirradiation in locoregional recurrence of rectal cancer. Sci. Rep..

[45] Isozaki Y., Yamada S., Kawashiro S., Yasuda S., Okada N., Ebner D., Tsuji H., Kamada T., Matsubara H. (2017). Carbon-ion radiotherapy for isolated para-aortic lymph node recurrence from colorectal cancer. J. Surg. Oncol..

[46] Matsuyama T., Yamauchi S., Masuda T., Kikuchi A., Tokunaga M., Sugihara K., Kinugasa Y. (2021). Treatment and subsequent prognosis in locally recurrent rectal cancer: A multicenter retrospective study of 498 patients. Int. J. Color. Dis..

[47] Murayama S., Yamada S., Hiroshima Y., Takiyama H., Taguchi H., Kimoto T., Anzai M., Hagiwara Y., Yasui K., Mori K. (2023). Particle beam therapy for pelvic recurrence of colorectal cancer: A registry data analysis in Japan and a systematic review. J. Radiat. Res..

[48] Yamada S., Kamada T., Kawashiro S., Isozaki Y., Ebner D.K. (2017). Update on Carbon-Ion Radiation Therapy for Patients With Pelvic Recurrence of Rectal Cancer. Int. J. Radiat. Oncol. Biol. Phys..

[49] Shirai K., Ohno T., Saitoh J., Okamoto M., Katoh H., Murata K., Kawamura H., Musha A., Abe T., Mizukami T. (2019). Prospective Study of Isolated Recurrent Tumor Re-irradiation With Carbon-Ion Beams. Front. Oncol..

[50] Hong T.S., Wo J.Y., Borger D.R., Yeap B.Y., McDonnell E.I., Willers H., Blaszkowsky L.S., Kwak E.L., Allen J.N., Clark J.W. (2017). Phase II Study of Proton-Based Stereotactic Body Radiation Therapy for Liver Metastases: Importance of Tumor Genotype. J. Natl. Cancer Inst..

[51] Kang J.I., Sufficool D.C., Hsueh C.T., Wroe A.J., Patyal B., Reeves M.E., Slater J.D., Yang G.Y. (2019). A phase I trial of Proton stereotactic body radiation therapy for liver metastases. J. Gastrointest. Oncol..

[52] Kim K., Yu J.I., Park H.C., Yoo G.S., Lim D., Noh J.M., Jeong W.K. (2022). A phase II trial of hypofractionated high-dose proton beam therapy for unresectable liver metastases. Radiother. Oncol..

[53] Yamaguchi H., Kato T., Honda M., Hamada K., Todate Y., Ishikawa Y., Seto I., Tominaga T., Machida M., Takagawa Y. (2023). Clinical outcomes and factors involved in the local control of proton beam therapy for oligometastatic liver tumors in patients with colorectal cancer. Strahlenther. Onkol..

[54] Makishima H., Yasuda S., Isozaki Y., Kasuya G., Okada N., Miyazaki M., Mohamad O., Matsufuji N., Yamada S., Tsuji H. (2019). Single fraction carbon ion radiotherapy for colorectal cancer liver metastasis: A dose escalation study. Cancer Sci..

[55] Shiba S., Wakatsuki M., Toyama S., Terashima K., Uchida H., Katoh H., Shibuya K., Okazaki S., Miyasaka Y., Ohno T. (2023). Carbon-ion radiotherapy for oligometastatic liver disease: A national multicentric study by the Japan Carbon-Ion Radiation Oncology Study Group (J-CROS). Cancer Sci..

[56] Shiba S., Shibuya K., Okamoto M., Okano N., Kubo N., Kaminuma T., Sato H., Okazaki S., Miyasaka Y., Kawamura H. (2021). Carbon-ion Radiotherapy for Oligometastatic Colorectal Cancer in the Liver or Lung. Anticancer. Res..

[57] Aibe N., Ogino H., Teramukai S., Yamazaki H., Iwata H., Matsuo Y., Okimoto T., Murakami M., Suzuki M., Arimura T. (2021). Multi-Institutional Retrospective Analysis of the Outcomes of Proton Beam Therapy for Patients With 1 to 3 Pulmonary Oligometastases From Various Primary Cancers. Adv. Radiat. Oncol..

[58] Takahashi W., Nakajima M., Yamamoto N., Yamada S., Yamashita H., Nakagawa K., Tsuji H., Kamada T. (2014). Carbon ion radiotherapy for oligo-recurrent lung metastases from colorectal cancer: A feasibility study. Radiat. Oncol..

[59] Okonogi N., Kaminuma T., Okimoto T., Shinoto M., Yamamoto N., Yamada S., Murata K., Ohno T., Shioyama Y., Tsuji H. (2019). Carbon-ion radiotherapy for lymph node oligo-recurrence: A multi-institutional study by the Japan Carbon-Ion Radiation Oncology Study Group (J-CROS). Int. J. Clin. Oncol..

[60] Lee J.J.B., Isozaki T., Choi S.H., Takiyama H., Lee J., Chang J.S., Yamada S., Koom W.S. (2025). Outcomes of Carbon-Ion Radiation Therapy Versus Photon Therapy for Isolated Paraortic Lymph Node Recurrence From Colorectal Cancer. Int. J. Radiat. Oncol. Biol. Phys..

[61] Aibe N., Ogino H., Wakatsuki M., Fujikawa K., Teramukai S., Fukumitsu N., Shiba S., Yamamoto N., Nomoto A., Ono T. (2023). Comprehensive analysis of Japanese nationwide cohort data of particle beam therapy for pulmonary, liver and lymph node oligometastases: Particle beam therapy versus high-precision X-ray radiotherapy. J. Radiat. Res..

[62] Fukumitsu N., Shiba S., Shibuya K., Kobayashi D., Miyasaka Y., Yamaguchi H., Numajiri H., Wakatsuki M., Ogino H., Katoh N. (2025). Comprehensive analysis of data from a nationwide Japanese cohort on particle therapy for metastatic liver tumors. Int. J. Radiat. Oncol. Biol. Phys..

[63] Czito B., Deming D., Jameson G., Mulcahy M., Vaghefi H., Dudley M., Holen K., DeLuca A., Mittapalli R., Munasinghe W. (2017). Safety and tolerability of veliparib combined with capecitabine plus radiotherapy in patients with locally advanced rectal cancer: A phase 1b study. Lancet Gastroenterol. Hepatol..

[64] Perez B., Aljumaily R., Marron T., Shafique M., Burris H., Iams W., Chmura S., Luke J., Edenfield W., Sohal D. (2024). Phase I study of peposertib and avelumab with or without palliative radiotherapy in patients with advanced solid tumors. ESMO Open.

[65] Parikh A.R., Szabolcs A., Allen J.N., Clark J.W., Wo J.Y., Raabe M., Thel H., Hoyos D., Mehta A., Arshad S. (2021). Radiation therapy enhances immunotherapy response in microsatellite stable colorectal and pancreatic adenocarcinoma in a phase II trial. Nat. Cancer.

[66] Xia F., Wang Y., Wang H., Shen L., Xiang Z., Zhao Y., Zhang H., Wan J., Zhang H., Wang Y. (2024). Randomized Phase II Trial of Immunotherapy-Based Total Neoadjuvant Therapy for Proficient Mismatch Repair or Microsatellite Stable Locally Advanced Rectal Cancer (TORCH). J. Clin. Oncol..

[67] Roxburgh C., Hanna C., Graham J., Saunders M., Samuel L., MacLeod N., Devlin L., Edwards J., Hillson L., McMahon R. (2023). Durvalumab (MEDI 4736) with extended neoadjuvant regimens in rectal cancer: A randomised phase II trial (PRIME-RT). J. Clin. Oncol..

[68] Roxburgh C., Hanna C., Saunders M., Arthur C., Samuel L., Wells L., Muirhead R., MacLeod N., Graham J., Devlin L. (2025). 1116 PRIME-RT: Durvalumab with extended neoadjuvant regimens in locally advanced rectal cancer (LARC): A randomised phase II trial. Radiother. Oncol..

[69] Lin Z.Y., Zhang P., Chi P., Xiao Y., Xu X.M., Zhang A.M., Qiu X.F., Wu J.X., Yuan Y., Wang Z.N. (2024). Neoadjuvant short-course radiotherapy followed by camrelizumab and chemotherapy in locally advanced rectal cancer (UNION): Early outcomes of a multicenter randomized phase III trial. Ann. Oncol..

[70] Zhang W., Tang Y., Wei L., Liu S., Wang W., Chi Y., Wang Y., Kang W., Huang W., Deng F. (2024). Preoperative short-course radiotherapy followed by chemotherapy and PD-1 inhibitor administration for locally advanced rectal cancer: A study protocol of a randomized phase II/III trial (STELLAR II study). Color. Dis..

[71] Tian F., Dai H., Sha D., Jing H., Li L., Jing C. (2025). Short-course radiotherapy followed by sintilimab and CAPOX as total neoadjuvant treatment in locally advanced rectal cancer: A prospective, randomized controlled trial (SPRING-01). J. Clin. Oncol..

[72] Shamseddine A., Zeidan Y., El Husseini Z., Kreidieh M., Al Darazi M., Turfa R., Kattan J., Khalifeh I., Mukherji D., Temraz S. (2020). Efficacy and safety-in analysis of short-course radiation followed by mFOLFOX-6 plus avelumab for locally advanced rectal adenocarcinoma. Radiat. Oncol..

[73] Wang F., Lai C., Lv Y., Zhang F., Shi L., Wang Y., Shen Y., Xu L., Hu P., Tang W. (2025). Efficacy and safety of combining short-course neoadjuvant chemoradiotherapy with envafolimab in locally advanced rectal cancer patients with microsatellite stability: A phase II PRECAM experimental study. Int. J. Surg..

[74] Rahma O., Yothers G., Hong T., Russell M., You Y.N., Parker W., Jacobs S., Colangelo L., Lucas P., Gollub M. (2021). Use of Total Neoadjuvant Therapy for Locally Advanced Rectal Cancer. JAMA Oncol..

[75] Bando H., Tsukada Y., Inamori K., Togashi Y., Koyama S., Kotani D., Fukuoka S., Yuki S., Komatsu Y., Homma S. (2022). Preoperative Chemoradiotherapy plus Nivolumab before Surgery in Patients with Microsatellite Stable and Microsatellite Instability-High Locally Advanced Rectal Cancer. Clin. Cancer Res..

[76] Grassi E., Zingaretti C., Petracci E., Corbelli J., Papiani G., Banchelli I., Valli I., Frassineti G.L., Passardi A., Di Bartolomeo M. (2023). Phase II study of capecitabine-based concomitant chemoradiation followed by durvalumab as a neoadjuvant strategy in locally advanced rectal cancer: The PANDORA trial. ESMO Open Cancer Horiz..

[77] Li Y., Pan C., Gao Y., Zhang L., Ji D., Cui X., Zhang X., Cai Y., Zhang Y., Yao Y. (2024). Total Neoadjuvant Therapy With PD-1 Blockade for High-Risk Proficient Mismatch Repair Rectal Cancer. JAMA Surg..

[78] Yang Z., Gao J., Zheng J., Han J., Li A., Liu G., Sun Y., Zhang J., Chen G., Xu R. (2024). Efficacy and safety of PD-1 blockade plus long-course chemoradiotherapy in locally advanced rectal cancer (NECTAR): A multi-center phase 2 study. Signal Transduct. Target. Ther..

[79] Xiao W.-W., Chen G., Gao Y.-H., Lin J.-Z., Wu X.-J., Luo H.-L., Lu Z.-H., Wang Q.-X., Sun R., Cai P.-Q. (2024). Effect of neoadjuvant chemoradiotherapy with or without PD-1 antibody sintilimab in pMMR locally advanced rectal cancer: A randomized clinical trial. Cancer Cell.

[80] Yang Y., Pang K., Lin G., Liu X., Gao J., Zhou J., Xu L., Gao Z., Wu Y., Li A. (2025). Neoadjuvant chemoradiation with or without PD-1 blockade in locally advanced rectal cancer: A randomized phase 2 trial. Nat. Med..

[81] Romesser P.B., Capdevila J., Garcia-Carbonero R., Philip T., Fernandez Martos C., Tuli R., Rodriguez-Gutierrez A., Kuipers M., Becker A., Coenen-Stass A. (2024). A Phase Ib Study of the DNA-PK Inhibitor Peposertib Combined with Neoadjuvant Chemoradiation in Patients with Locally Advanced Rectal Cancer. Clin. Cancer Res..

[82] Tian F., Dai H., Sha D., Yu Y., Jing H., Sun C., Shang L., Liu Y., Feng R., Li J. (2025). Total neoadjuvant treatment with short-course radiotherapy followed by sintilimab plus capecitabine–oxaliplatin versus short-course radiotherapy followed by capecitabine–oxaliplatin in patients with locally advanced rectal cancer (SPRING-01): A single-centre, open-label, phase 2, randomised controlled trial. Lancet Oncol..

[83] Tang Y., Li H.-Y., Wei L.-C., Li N., Zhang W.-J., Lu Y.-F., Deng F.-Y., Xu T.-Z., Shuai J.-C., Lei Z.-F. (2025). Short-course-based TNT with or without PD-1 inhibitor for pMMR locally advanced rectal cancer: Phase 2 results of a randomized trial (STELLAR II). Med.

