# Supplementary material for: Clinical Evidence on Particle Radiation, DNA Damage Response Inhibitors, and Immunotherapy for Mismatch Repair-Proficient Rectal Cancer
Source: Cancers (Basel). 2026 Feb 17;18(4):652. doi: 10.3390/cancers18040652 (PMC12939555; doi:10.3390/cancers18040652)
Supplement: Supplementary file 1 [file cancers-18-00652-s001.zip › cancers-4127889-non-published.pdf]

a. **MEDLINE**

Search terms: ((("rectal cancer"[Text Word] OR "colorectal cancer"[Text Word]) OR ("rectal carcinoma"[Text Word] OR "colorectal carcinoma"[Text Word]) OR ("rectal neoplasm"[Text Word] OR "colorectal neoplasm"[Text Word]) OR ("rectal tumor"[Text Word] OR "colorectal tumor"[Text Word]) OR ("colorectal cells"[Text Word])) AND (("high LET radiation"[Text Word] OR "high linear energy transfer radiation"[Text Word]) OR ("proton therapy"[Text Word] OR "protons"[Text Word]) OR ("carbon ion radiation therapy"[Text Word] OR "CIRT"[Text Word]) OR ("diffusing alpha particle radiation therapy"[Text Word] OR "DaRT"[Text Word]))) AND ("2014/01/01"[PubDate] : "2024/12/31"[PubDate]))

For studies involving combinations with immunotherapy or DDR inhibitors: ((("rectal cancer"[Text Word] OR "colorectal cancer"[Text Word]) OR ("rectal carcinoma"[Text Word] OR "colorectal carcinoma"[Text Word]) OR ("rectal neoplasm"[Text Word] OR "colorectal neoplasm"[Text Word]) OR ("rectal tumor"[Text Word] OR "colorectal tumor"[Text Word]) OR ("colorectal cells"[Text Word])) AND (("high LET radiation"[Text Word] OR "high linear energy transfer radiation"[Text Word]) OR ("proton therapy"[Text Word] OR "protons"[Text Word]) OR ("carbon ion radiation therapy"[Text Word] OR "CIRT"[Text Word]) OR ("diffusing alpha particle radiation therapy"[Text Word] OR "DaRT"[Text Word]) OR ("conventional X-ray radiation therapy"[Text Word] AND ("immunotherapy"[Text Word] OR ("PD-1"[Text Word] OR "PD-L1"[Text Word])) OR ("DDR inhibitors"[Text Word] OR "PARP inhibitors"[Text Word] OR "ATR inhibitors"[Text Word] OR "ATM inhibitors"[Text Word] OR "CHK1 inhibitors"[Text Word] OR "CHK2 inhibitors"[Text Word]))))) AND ("2014/01/01"[PubDate] : "2024/12/31"[PubDate])

b. **PubMed**

Search terms: For studies on high LET radiation alone: ("rectal cancer"[Title/Abstract] OR "colorectal cancer"[Title/Abstract] OR "rectal carcinoma"[Title/Abstract] OR "colorectal carcinoma"[Title/Abstract] OR "rectal neoplasm"[Title/Abstract] OR "colorectal neoplasm"[Title/Abstract] OR "rectal tumor"[Title/Abstract] OR "colorectal tumor"[Title/Abstract] OR "colorectal cells"[Title/Abstract]) AND ("high LET radiation"[Title/Abstract] OR "high linear energy transfer radiation"[Title/Abstract] OR "proton therapy"[Title/Abstract] OR "proton radiation"[Title/Abstract] OR "proton irradiation"[Title/Abstract] OR "protons"[Title/Abstract] OR "carbon ion"[Title/Abstract] OR "CIRT"[Title/Abstract] OR "diffusing alpha-emitters"[Title/Abstract] OR "diffusing alpha emitters"[Title/Abstract] OR "alpha radiation"[Title/Abstract] OR "alpha particles"[Title/Abstract) AND 2014/01/01:2025/01/01[Date - Publication]

For studies involving combinations with immunotherapy or DDR inhibitors: ((("rectal cancer"[Title/Abstract] OR "colorectal cancer"[Title/Abstract]) OR ("rectal carcinoma"[Title/Abstract] OR "colorectal carcinoma"[Title/Abstract]) OR ("rectal neoplasm"[Title/Abstract] OR "colorectal neoplasm"[Title/Abstract]) OR ("rectal

tumor"[Title/Abstract] OR "colorectal tumor"[Title/Abstract]) OR ("colorectal cells"[Title/Abstract])) AND (("high LET radiation"[Title/Abstract] OR "high linear energy transfer radiation"[Title/Abstract]) OR ("proton therapy"[Title/Abstract] OR "protons"[Title/Abstract]) OR ("carbon ion radiation therapy"[Title/Abstract] OR "CIRT"[Title/Abstract]) OR ("diffusing alpha particle radiation therapy"[Title/Abstract] OR "DaRT"[Title/Abstract]) OR ("conventional X-ray radiation therapy"[Title/Abstract] AND ("immunotherapy"[Title/Abstract] OR ("PD-1"[Title/Abstract] OR "PDL1"[Title/Abstract] OR ("DDR inhibitors"[Title/Abstract] OR "PARP inhibitors"[Title/Abstract] OR "ATR inhibitors"[Title/Abstract] OR "ATM inhibitors"[Title/Abstract] OR "CHK1 inhibitors"[Title/Abstract] OR "CHK2 inhibitors"[Title/Abstract]))))) AND ("2014/01/01"[PDAT] : "2025/01/01"[PDAT])

c. **Google Scholar**
Search strategy:
• In the title of the article: (intitle:"rectal cancer" OR intitle:"colorectal cancer" OR intitle:"rectal carcinoma" OR intitle:"colorectal carcinoma" OR intitle:"rectal tumor" OR intitle:"colorectal tumor" OR intitle:"rectal neoplasm" OR intitle:"colorectal neoplasm" OR intitle:"colorectal cells") AND (intitle:"high LET radiation" OR intitle:"high linear energy transfer radiation" OR intitle:"proton therapy" OR intitle:"proton irradiation" OR intitle:"proton radiation" OR intitle:"protons" OR intitle:"carbon ion" OR intitle:"CIRT" OR intitle:"diffusing alpha-emitters" OR intitle:"diffusing alpha emitters" OR intitle:"alpha radiation" OR intitle:"alpha particles")
• No additional filters applied for studies including high LET radiation alone.
• For combination studies: With at least one of the words: o "PD-1" OR "PD-L1" OR "checkpoint blockade" OR "immunotherapy" OR "PARP inhibitors" OR "DDR inhibitors" OR "ATM inhibitors" OR "ATR inhibitors" OR "CHK1 inhibitors" OR "CHK2 inhibitors"
• Filters: o Exclude patents and citations o Date range: 2014–2025

d. **Cochrane Library**
Search terms: (("rectal cancer":ti,ab,kw OR "colorectal cancer":ti,ab,kw OR "colorectal cells":ti,ab,kw) AND ("high LET radiation":ti,ab,kw OR "proton therapy":ti,ab,kw OR "protons":ti,ab,kw OR "proton radiation":ti,ab,kw OR "proton irradiation":ti,ab,kw OR "CIRT":ti,ab,kw OR "carbon ion":ti,ab,kw OR "diffusing alpha":ti,ab,kw OR "alpha particles":ti,ab,kw OR "alpha radiation":ti,ab,kw OR "DaRT":ti,ab,kw))"
For combination studies: (("rectal cancer":ti,ab,kw OR "colorectal cancer":ti,ab,kw OR "rectal carcinoma":ti,ab,kw OR "colorectal carcinoma":ti,ab,kw OR "rectal tumor":ti,ab,kw OR "colorectal tumor":ti,ab,kw OR "rectal neoplasm":ti,ab,kw OR "colorectal neoplasm":ti,ab,kw OR "colorectal cells":ti,ab,kw) AND ("high LET radiation":ti,ab,kw OR "proton therapy":ti,ab,kw OR "protons":ti,ab,kw OR "proton radiation":ti,ab,kw OR "proton irradiation":ti,ab,kw OR "CIRT":ti,ab,kw OR "carbon ion":ti,ab,kw OR "diffusing alpha":ti,ab,kw OR "alpha particles":ti,ab,kw OR "alpha

radiation":ti,ab,kw OR "DaRT":ti,ab,kw OR "conventional radiotherapy":ti,ab,kw OR "X-ray radiation":ti,ab,kw OR "photon therapy":ti,ab,kw OR "external beam radiation":ti,ab,kw OR "photon radiation":ti,ab,kw OR "XRT":ti,ab,kw OR "IMRT":ti,ab,kw OR "3D conformal radiation therapy":ti,ab,kw))
AND ("immunotherapy":ti,ab,kw OR "PD-1":ti,ab,kw OR "PD-L1":ti,ab,kw OR "checkpoint blockade":ti,ab,kw OR "DDR inhibitors":ti,ab,kw OR "PARP inhibitors":ti,ab,kw OR "ATR inhibitors":ti,ab,kw OR "ATM inhibitors":ti,ab,kw OR "CHK1 inhibitors":ti,ab,kw OR "CHK2 inhibitors":ti,ab,kw))
Search settings: • Date range: 2014–2025

e. **Web of Science**
TS=("rectal cancer" OR "colorectal cancer" OR "rectal carcinoma" OR "colorectal carcinoma" OR "rectal tumor" OR "colorectal tumor" OR "rectal neoplasm" OR "colorectal neoplasm" OR "colorectal cells") AND
TS=("high LET radiation" OR "high linear energy transfer radiation" OR "proton therapy" OR "proton irradiation" OR "proton radiation" OR "protons" OR "carbon ion" OR "CIRT" OR "diffusing alpha-emitters" OR "diffusing alpha emitters" OR "alpha radiation" OR "alpha particles")
PY=(2014-2025)
For combination studies:
TS=("rectal cancer" OR "colorectal cancer" OR "rectal carcinoma" OR "colorectal carcinoma" OR "rectal tumor" OR "colorectal tumor" OR "rectal neoplasm" OR "colorectal neoplasm" OR "colorectal cells")
AND
TS=("high LET radiation" OR "high linear energy transfer radiation" OR "proton therapy" OR "proton radiation" OR "proton irradiation" OR "protons" OR "carbon ion" OR "CIRT" OR "diffusing alpha emitters" OR "DaRT" OR "diffusing alpha particle radiation therapy" OR "carbon ion radiation therapy"
OR "conventional radiotherapy" OR "X-ray radiation" OR "photon therapy" OR "conventional radiation" OR "external beam radiation" OR "photon radiation" OR "XRT" OR "IMRT" OR "3D conformal radiation therapy")
AND
TS=("immunotherapy" OR "PD-1" OR "PD-L1" OR "checkpoint blockade" OR "DDR inhibitors" OR "PARP inhibitors" OR "ATR inhibitors" OR "ATM inhibitors" OR "CHK1 inhibitors" OR "CHK2 inhibitors")
AND
PY=(2014-2025)

f. Epistemonikos
Search terms: (title:(("rectal cancer" OR "colorectal cancer" OR "rectal carcinoma" OR "colorectal carcinoma") AND ("high LET radiation" OR "high linear energy transfer radiation" OR "proton therapy" OR "proton irradiation" OR "proton radiation" OR "protons" OR "carbon ion" OR "CIRT" OR "diffusing alpha-emitters"

OR "diffusing alpha emitters" OR "alpha radiation" OR "alpha particles")) OR abstract:(("rectal cancer" OR "colorectal cancer" OR "rectal carcinoma" OR "colorectal carcinoma") AND ("high LET radiation" OR "high linear energy transfer radiation" OR "proton therapy" OR "proton irradiation" OR "proton radiation" OR "protons" OR "carbon ion" OR "CIRT" OR "diffusing alpha-emitters" OR "diffusing alpha emitters" OR "alpha radiation" OR "alpha particles")))

Combination studies: title:(("rectal cancer" OR "colorectal cancer" OR "rectal carcinoma" OR "colorectal carcinoma" OR "rectal tumor" OR "colorectal tumor" OR "rectal neoplasm" OR "colorectal neoplasm") AND ("high LET radiation" OR "high linear energy transfer radiation" OR "proton therapy" OR "proton irradiation" OR "proton radiation" OR "protons" OR "carbon ion" OR "CIRT" OR "diffusing alpha emitters" OR "DaRT" OR "alpha radiation" OR "alpha particles" OR "conventional radiotherapy" OR "X-ray radiation" OR "photon therapy" OR "proton beam therapy" OR "external beam radiation" OR "photon radiation" OR "XRT" OR "IMRT" OR "3D conformal radiation therapy")) OR abstract:(("rectal cancer" OR "colorectal cancer" OR "rectal carcinoma" OR "colorectal carcinoma" OR "rectal tumor" OR "colorectal tumor" OR "rectal neoplasm" OR "colorectal neoplasm") AND ("high LET radiation" OR "high linear energy transfer radiation" OR "proton therapy" OR "proton irradiation" OR "proton radiation" OR "protons" OR "carbon ion" OR "CIRT" OR "diffusing alpha emitters" OR "DaRT" OR "alpha radiation" OR "alpha particles" OR "conventional radiotherapy" OR "X-ray radiation" OR "photon therapy" OR "proton beam therapy" OR "external beam radiation" OR "photon radiation" OR "XRT" OR "IMRT" OR "3D conformal radiation therapy"))) AND (title:("immunotherapy" OR "PD-1" OR "PD-L1" OR "DDR inhibitors" OR "PARP inhibitors" OR "ATR inhibitors" OR "ATM inhibitors" OR "CHK1 inhibitors" OR "CHK2 inhibitors") OR abstract:("immunotherapy" OR "PD-1" OR "PD-L1" OR "DDR inhibitors" OR "PARP inhibitors" OR "ATR inhibitors" OR "ATM inhibitors" OR "CHK1 inhibitors" OR "CHK2 inhibitors"))

Search settings: • Date range: 2014–2025
