# Supplementary material for: Clinical Evidence on Particle Radiation, DNA Damage Response Inhibitors, and Immunotherapy for Mismatch Repair-Proficient Rectal Cancer
Source: Cancers (Basel). 2026 Feb 17;18(4):652. doi: 10.3390/cancers18040652 (PMC12939555; doi:10.3390/cancers18040652)
Supplement: Supplementary file 1 [file cancers-18-00652-s001.zip › cancers-4127889-supplementary.pdf]

I. PICO search strategy:

Table 1. Population, Intervention, Control, Outcome, Study Design (PICOS) inclusion criteria for high-LET radiation and/or combination studies	
**Population**	Patients diagnosed with MSS CRC, or unknown status.
**Intervention**	Received PBT, CIRT or DaRT, either alone or combined with ICI or DDRi, or XRT combined with ICI and DDRi.
**Control**	Control group was not necessary. Non-high-LET RT, if any. For combination treatments, comparison with monotherapies.
**Outcomes**	The study must report at least one of the following outcomes for CRC patients: overall survival rate, local control rate, complete response rate, progression free survival rate; tumor volume response for preclinical.
**Study design**	Clinical trials, prospective, and retrospective studies were eligible. Case reports, reviews and in silico studies were excluded. Abstracts and preprints evidence conditionally accepted, if no articles referred to the topic.

II. Database search:

Last search 8/31/2025.
Date range: 01/01/2014-08/31/2025

a. **Scopus:**

1) High-LET search strategy:

TITLE-ABS-KEY("rectal cancer" OR "colorectal cancer" OR "rectal carcinoma" OR "colorectal carcinoma" OR "rectal tumor" OR "colorectal tumor" OR "rectal neoplasm" OR "colorectal neoplasm" OR "colorectal cells"
AND
"high LET radiation" OR "high linear energy transfer radiation" OR "proton therapy" OR "proton irradiation" OR "proton radiation" OR "proton beam" OR "IMPT" OR "carbon ion" OR "CIRT" OR "diffusing alpha-emitters" OR "diffusing alpha emitters" OR "alpha radiation" OR "alpha particles")

2) Combination therapies search strategy:

TITLE-ABS-KEY("rectal cancer" OR "colorectal cancer" OR "rectal carcinoma" OR "colorectal carcinoma" OR "rectal tumor" OR "colorectal tumor" OR "rectal neoplasm" OR "colorectal neoplasm" OR "colorectal cells"
AND
"high LET radiation" OR "high linear energy transfer radiation" OR "proton therapy" OR "proton irradiation" OR "proton radiation" OR "proton beam" OR "IMPT" OR "carbon ion" OR "CIRT" OR "diffusing alpha emitters" OR "DaRT" OR "diffusing alpha particle radiation therapy" OR "conventional radiotherapy" OR "X-ray radiation" OR "photon therapy" OR "conventional radiation" OR "external beam radiation" OR "photon radiation" OR "XRT" OR "IMRT" OR "3D conformal radiation therapy"
AND

"immune checkpoint blockade" OR "immune checkpoint inhibitor" OR "PD-1" OR "PD-L1" OR "CTLA4" OR "DNA damage response" OR "PARP" OR "Poly (ADP-ribose) polymerase" OR "DNA-PK" OR "DNA-dependent protein kinase" OR "ATR" OR "Ataxia telangiectasia and Rad3-related protein" OR "WEE1" OR "WEE1 G2 checkpoint kinase" OR "Rad51" OR "WEE1 G2 checkpoint kinase" OR "ATM" OR "Ataxia telangiectasia mutated" OR "CHK1" OR "Checkpoint kinase 1" OR "CHK2" OR "Checkpoint kinase 2")

b. Epistemonikos:

1) High-LET search strategy:

((title:("rectal cancer" OR "colorectal cancer" OR "rectal carcinoma" OR "colorectal carcinoma" OR "rectal tumor" OR "colorectal tumor" OR "rectal neoplasm" OR "colorectal neoplasm" OR "colorectal cells")
OR
abstract:("rectal cancer" OR "colorectal cancer" OR "rectal carcinoma" OR "colorectal carcinoma" OR "rectal tumor" OR "colorectal tumor" OR "rectal neoplasm" OR "colorectal neoplasm" OR "colorectal cells"))
AND
(title:("high LET radiation" OR "high linear energy transfer radiation" OR "proton therapy" OR "proton irradiation" OR "proton radiation" OR "proton beam" OR "IMPT" OR "carbon ion" OR "CIRT" OR "diffusing alpha-emitters" OR "diffusing alpha emitters" OR "alpha radiation" OR "alpha particles")
OR
abstract:("high LET radiation" OR "high linear energy transfer radiation" OR "proton therapy" OR "proton irradiation" OR "proton radiation" OR "proton beam" OR "IMPT" OR "carbon ion" OR "CIRT" OR "diffusing alpha-emitters" OR "diffusing alpha emitters" OR "alpha radiation" OR "alpha particles")))

2) Combination therapies search strategy:

((title:("immune checkpoint blockade" OR "immune checkpoint inhibitor" OR "PD-1" OR "PD-L1" OR "CTLA4" OR "DNA damage response" OR "PARP" OR "Poly (ADP-ribose) polymerase" OR "DNA-PK" OR "DNA-dependent protein kinase" OR "ATR" OR "Ataxia telangiectasia Rad3-related protein" OR "WEE1" OR "WEE1 G2 checkpoint kinase" OR "Rad51" OR "ATM" OR "Ataxia telangiectasia mutated" OR "CHK1" OR "Checkpoint kinase 1" OR "CHK2" OR "Checkpoint kinase 2")
OR
abstract:("immune checkpoint blockade" OR "immune checkpoint inhibitor" OR "PD-1" OR "PD-L1" OR "CTLA4" OR "DNA damage response" OR "PARP" OR "Poly (ADP-ribose) polymerase" OR "DNA-PK" OR "DNA-dependent protein kinase" OR "ATR" OR "Ataxia telangiectasia Rad3-related protein" OR "WEE1" OR "WEE1 G2 checkpoint kinase" OR "Rad51" OR "ATM" OR "Ataxia telangiectasia mutated" OR "CHK1" OR "Checkpoint kinase 1" OR "CHK2" OR "Checkpoint kinase 2"))
AND
(title:("rectal cancer" OR "colorectal cancer" OR "rectal carcinoma" OR "colorectal carcinoma" OR "rectal tumor" OR "colorectal tumor" OR "rectal neoplasm" OR "colorectal neoplasm" OR "colorectal cells")
OR
abstract:("rectal cancer" OR "colorectal cancer" OR "rectal carcinoma" OR "colorectal carcinoma" OR "rectal tumor" OR "colorectal tumor" OR "rectal neoplasm" OR "colorectal neoplasm" OR "colorectal cells"))
AND

(title:("high LET radiation" OR "high linear energy transfer radiation" OR "proton therapy" OR "proton irradiation" OR "proton radiation" OR "proton beam" OR "IMPT" OR "carbon ion" OR "CIRT" OR "diffusing alpha emitters" OR "DaRT" OR "diffusing alpha particle radiation therapy" OR "carbon ion radiation therapy" OR "conventional radiotherapy" OR "X-ray radiation" OR "photon therapy" OR "conventional radiation" OR "external beam radiation" OR "photon radiation" OR "XRT" OR "IMRT" OR "3D conformal radiation therapy")
OR
abstract:("high LET radiation" OR "high linear energy transfer radiation" OR "proton therapy" OR "proton irradiation" OR "proton radiation" OR "proton beam" OR "IMPT" OR "carbon ion" OR "CIRT" OR "diffusing alpha emitters" OR "DaRT" OR "diffusing alpha particle radiation therapy" OR "carbon ion radiation therapy" OR "conventional radiotherapy" OR "X-ray radiation" OR "photon therapy" OR "conventional radiation" OR "external beam radiation" OR "photon radiation" OR "XRT" OR "IMRT" OR "3D conformal radiation therapy")))

c. PubMed Central

1) High-LET search strategy:

("rectal cancer"[Title/Abstract] OR "colorectal cancer"[Title/Abstract] OR "rectal adenocarcinoma"[Title/Abstract] OR "colorectal adenocarcinoma"[Title/Abstract] OR "rectal carcinoma"[Title/Abstract] OR "colorectal carcinoma"[Title/Abstract] OR "rectal neoplasm"[Title/Abstract] OR "colorectal neoplasm"[Title/Abstract] OR "rectal tumor"[Title/Abstract] OR "colorectal tumor"[Title/Abstract] OR "rectal malignancy"[Title/Abstract] OR "colorectal malignancy"[Title/Abstract]) AND ("high LET radiation" OR "high linear energy transfer radiation" OR "proton-therapy" OR "proton beam" OR "proton therapy" OR "proton irradiation" OR "proton radiation" OR "proton beam" OR "IMPT" OR "carbon ion radiation therapy" OR "CIRT" OR "diffusing alpha particle radiation therapy"[tiab:~0] OR "DaRT")

2) Combination therapies search strategy:

(("rectal cancer"[Title/Abstract] OR "colorectal cancer"[Title/Abstract] OR "rectal carcinoma"[Title/Abstract] OR "colorectal carcinoma"[Title/Abstract] OR "rectal tumor"[Title/Abstract] OR "colorectal tumor"[Title/Abstract] OR "rectal neoplasm"[Title/Abstract] OR "colorectal neoplasm"[Title/Abstract] OR "colorectal cells"[Title/Abstract]) AND ("high LET radiation"[Title/Abstract] OR "high linear energy transfer radiation"[Title/Abstract] OR "proton therapy"[Title/Abstract] OR "proton irradiation"[Title/Abstract] OR "proton radiation"[Title/Abstract] OR "proton beam"[Title/Abstract] OR "IMPT"[Title/Abstract] OR "carbon ion"[Title/Abstract] OR "CIRT"[Title/Abstract] OR "diffusing alpha emitters"[Title/Abstract] OR "DaRT"[Title/Abstract] OR "diffusing alpha particle radiation therapy"[Title/Abstract] OR "conventional radiotherapy"[Title/Abstract] OR "X-ray radiation"[Title/Abstract] OR "photon therapy"[Title/Abstract] OR "conventional radiation"[Title/Abstract] OR "external beam radiation"[Title/Abstract] OR "photon radiation"[Title/Abstract] OR "XRT"[Title/Abstract] OR "IMRT"[Title/Abstract] OR "3D conformal radiation therapy"[Title/Abstract])) AND ("immune checkpoint blockade"[Title/Abstract] OR "immune checkpoint inhibitor"[Title/Abstract] OR "PD-1"[Title/Abstract] OR "PD-L1"[Title/Abstract] OR "CTLA4"[Title/Abstract] OR "DNA damage response"[Title/Abstract] OR "PARP"[Title/Abstract] OR "Poly (ADP-ribose) polymerase"[Title/Abstract] OR "DNA-PK"[Title/Abstract] OR "DNA-dependent protein kinase"[Title/Abstract] OR "ATR"[Title/Abstract] OR "Ataxia telangiectasia and Rad3-related protein"[Title/Abstract] OR "WEE1"[Title/Abstract] OR "WEE1 G2 checkpoint

kinase"[Title/Abstract] OR "Rad51"[Title/Abstract] OR "WEE1 G2 checkpoint kinase"[Title/Abstract] OR "ATM"[Title/Abstract] OR "Ataxia telangiectasia mutated"[Title/Abstract] OR "CHK1"[Title/Abstract] OR "Checkpoint kinase 1"[Title/Abstract] OR "CHK2"[Title/Abstract] OR "Checkpoint kinase 2"[Title/Abstract])

d. Cochrane Library

1) High-LET search strategy:

("rectal cancer" OR "colorectal cancer" OR "rectal carcinoma" OR "colorectal carcinoma" OR "rectal tumor" OR "colorectal tumor" OR "rectal neoplasm" OR "colorectal neoplasm" OR "colorectal cells"):ti,ab,kw AND
("high LET radiation" OR "high linear energy transfer radiation" OR "proton therapy" OR "proton irradiation" OR "proton radiation" OR "proton beam" OR "IMPT" OR "carbon ion" OR "CIRT" OR "diffusing alpha-emitters" OR "diffusing alpha emitters" OR "alpha radiation" OR "alpha particles"):ti,ab,kw

2) Combination therapies search strategy:

("rectal cancer" OR "colorectal cancer" OR "rectal carcinoma" OR "colorectal carcinoma" OR "rectal tumor" OR "colorectal tumor" OR "rectal neoplasm" OR "colorectal neoplasm" OR "colorectal cells"):ti,ab,kw AND ("high LET radiation" OR "high linear energy transfer radiation" OR "proton therapy" OR "proton radiation" OR "proton irradiation" OR "protons" OR "carbon ion" OR "CIRT" OR "diffusing alpha emitters" OR "DaRT" OR "diffusing alpha particle radiation therapy" OR "carbon ion radiation therapy" OR "conventional radiotherapy" OR "X-ray radiation" OR "photon therapy" OR "conventional radiation" OR "external beam radiation" OR "photon radiation" OR "XRT" OR "IMRT" OR "3D conformal radiation therapy"):ti,ab,kw AND ("immune checkpoint blockade" OR "immune checkpoint inhibitor" OR "PD-1" OR "PD-L1" OR "CTLA4" OR "DNA damage response" OR "PARP" OR "Poly (ADP-ribose) polymerase" OR "DNA-PK" OR "DNA-dependent protein kinase" OR "ATR" OR "Ataxia telangiectasia and Rad3-related protein" OR "WEE1" OR "WEE1 G2 checkpoint kinase" OR "Rad51" OR "ATM" OR "Ataxia telangiectasia mutated" OR "CHK1" OR "Checkpoint kinase 1" OR "CHK2" OR "Checkpoint kinase 2"):ti,ab,kw

e. Web of Science:

1) High-LET search strategy:

TS=("rectal cancer" OR "colorectal cancer" OR "rectal carcinoma" OR "colorectal carcinoma" OR "rectal tumor" OR "colorectal tumor" OR "rectal neoplasm" OR "colorectal neoplasm" OR "colorectal cells") AND
TS=("high LET radiation" OR "high linear energy transfer radiation" OR "proton therapy" OR "proton irradiation" OR "proton radiation" OR "proton beam" OR "IMPT" OR "carbon ion" OR "CIRT" OR "diffusing alpha-emitters" OR "diffusing alpha emitters" OR "alpha radiation" OR "alpha particles")

2) Combination therapies search strategy:

TS=("rectal cancer" OR "colorectal cancer" OR "rectal carcinoma" OR "colorectal carcinoma" OR "rectal tumor" OR "colorectal tumor" OR "rectal neoplasm" OR "colorectal neoplasm" OR "colorectal cells")
AND
TS=("high LET radiation" OR "high linear energy transfer radiation" OR "proton therapy" OR "proton irradiation" OR "proton radiation" OR "proton beam" OR "IMPT" OR "carbon ion" OR "CIRT" OR "diffusing alpha emitters" OR "DaRT" OR "diffusing alpha particle radiation therapy" OR "carbon ion radiation therapy" OR "conventional radiotherapy" OR "X-ray radiation" OR "photon therapy" OR "conventional radiation" OR "external beam

radiation" OR "photon radiation" OR "XRT" OR "IMRT" OR "3D conformal radiation therapy")
AND
TS=("immune checkpoint blockade" OR "immune checkpoint inhibitor" OR "PD-1" OR "PD-L1" OR "CTLA4" OR "DNA damage response" OR "PARP" OR "Poly (ADP-ribose) polymerase" OR "DNA-PK" OR "DNA-dependent protein kinase" OR "ATR" OR "Ataxia telangiectasia and Rad3-related protein" OR "WEE1" OR "WEE1 G2 checkpoint kinase" OR "Rad51" OR "WEE1 G2 checkpoint kinase" OR "ATM" OR "Ataxia telangiectasia mutated" OR "CHK1" OR "Checkpoint kinase 1" OR "CHK2" OR "Checkpoint kinase 2")

f. Other sources: Google Scholar

1) High-LET search strategy:

allintitle:("rectal cancer" OR "colorectal cancer" OR "rectal carcinoma" OR "colorectal carcinoma" OR "rectal tumor" OR "colorectal tumor" OR "rectal neoplasm" OR "colorectal neoplasm" OR "colorectal cells") AND ("high LET radiation" OR "high linear energy transfer radiation" OR "proton therapy" OR "proton irradiation" OR "proton radiation" OR "proton beam" OR IMPT OR "carbon ion" OR CIRT OR "diffusing alpha-emitters" OR "diffusing alpha emitters" OR "alpha radiation" OR "alpha particles")

2) Combination therapies search strategy:

allintitle:("rectal cancer" OR "colorectal cancer" OR "rectal carcinoma" OR "colorectal carcinoma" OR "rectal tumor" OR "colorectal tumor" OR "rectal neoplasm" OR "colorectal neoplasm" OR "colorectal cells") AND ("high LET radiation" OR "high linear energy transfer radiation" OR "proton therapy" OR "proton irradiation" OR "proton radiation" OR "proton beam" OR IMPT OR "carbon ion" OR CIRT OR "diffusing alpha emitters" OR DaRT OR "diffusing alpha particle radiation therapy" OR "conventional radiotherapy" OR "X-ray radiation" OR "photon therapy" OR "conventional radiation" OR "external beam radiation" OR "photon radiation" OR XRT OR IMRT OR "3D conformal radiation therapy") AND ("immune checkpoint blockade" OR "immune checkpoint inhibitor" OR "PD-1" OR "PD-L1" OR CTLA4 OR "DNA damage response" OR PARP OR "Poly (ADP-ribose) polymerase" OR "DNA-PK" OR "DNA-dependent protein kinase" OR ATR OR "Ataxia telangiectasia and Rad3-related protein" OR WEE1 OR "WEE1 G2 checkpoint kinase" OR Rad51 OR ATM OR "Ataxia telangiectasia mutated" OR CHK1 OR "Checkpoint kinase 1" OR CHK2 OR "Checkpoint kinase 2")

III. MSS/pMMR studies identified by searching:

("pMMR" OR "proficient mismatch repair" OR "MMR proficiency" OR "mismatch repair proficiency" OR "microsatellite stable" OR "MSS")
OR
("CT26" OR "Colon26" OR "MC26" OR "SL4" OR "APCmin/+" OR "LuM-1" OR "NM11" OR "C106" OR "C125PM" OR "C32" OR "C70" OR "C75" OR "C80" OR "C84" OR "C99" OR "CACO2" OR "CAR1" OR "CL-11" OR "CL-14" OR "CL-40" OR "COCM1" OR "COGA2" OR "COGA5" OR "COGA5L" OR "COGA8" OR "Colo201" OR "Colo205" OR "Colo206" OR "Colo320" OR "Colo320DM" OR "Colo320HSR" OR "Colo678" OR "Colo741" OR "Colo94H" OR "CX1" OR "DIFI" OR "EB" OR "FET" OR "FRI" OR "HCA24" OR "HCA46" OR "HCC2998" OR "HDC114" OR "HDC142" OR "HDC54" OR "HDC8" OR "HDC82" OR "HRA16" OR "HRA19" OR "HROC18" OR

"HROC32" OR "HROC39" OR "HROC46" OR "HROC69" OR "HROC80" OR "HT115" OR "HT29" OR "HT55" OR "HUTU80" OR "IS1" OR "IS3" OR "KM20" OR "LIM1863" OR "LIM2099" OR "LIM2405" OR "LS1034" OR "LS123" OR "LS513" OR "MDST8" OR "NCI-H498" OR "NCI-H508" OR "NCI-H684" OR "NCI-H716" OR "OUMS23" OR "OXCO1" OR "OXCO3" OR "PCJW" OR "RCM1" OR "RW2982" OR "RW7213" OR "SCKO-1" OR "SK-CO-1" OR "SKCO-1" OR "SNU1033" OR "SNU1181" OR "SNU1235" OR "SNU1411" OR "SNU1460" OR "SNU254" OR "SNU283" OR "SNU479" OR "SNU503" OR "SNU61" OR "SNU81" OR "SNU977" OR "SNUC1" OR "SW1116" OR "SW1222" OR "SW1417" OR "SW1463" OR "SW403" OR "SW480" OR "SW620" OR "SW837" OR "SW948" OR "T84" OR "V411" OR "V9P" OR "VACO10MS" OR "VACO4A" OR "VACO4S" OR "WiDr")
